# USEtox modeling of children’s exposures to Bisphenol A (BPA) and alternatives in toys

**DOI:** 10.1038/s41370-025-00827-6

**Published:** 2026-02-12

**Authors:** Lei Huang, Lynn Nakayama Wong, Xiaoying Zhou, Michelle Romero-Franco, Nathalie Pham, Hyeong-Moo Shin, Thomas McKone, Qingyu Meng, Olivier Jolliet

**Affiliations:** 1 California Department of Toxic Substances Control, Sacramento, CA USA; 2 Department of Environmental Health Sciences, School of Public Health, University of Michigan, Ann Arbor, MI USA; 3 Department of Environmental Science, Baylor University, Waco, TX USA; 4 Division of Environmental Health Sciences, School of Public Health, University of California, Berkeley, CA USA; 5 Quantitative Sustainability Assessment, Department of Environmental and Resource Engineering, Technical University of Denmark, 2800 Kgs, Lyngby, Denmark

**Keywords:** Endocrine disruptors, Aggregate exposure, Exposure modeling, Children exposure/health, Chemicals in products

## Abstract

**Background:**

Bisphenol A (BPA) is a known endocrine disruptor, raising concerns about its presence in children’s toys. Despite its well-studied health effects, there is limited research addressing aggregate exposure to BPA in toys. Moreover, hazard and exposure information on BPA alternatives on the market is scarce.

**Objective:**

This study applies USEtox modeling to systematically evaluate young children’s exposure to BPA in toys and to compare the exposure between BPA and potential alternatives.

**Methods:**

We assessed the exposure to BPA and alternatives across representative toy archetypes and identified dominant exposure pathways and key influencing factors. We also estimated aggregate exposure for 3–6-year-olds and evaluated health risks by comparing exposure doses to toxicity benchmarks. We assumed a mass fraction of 300 ppm of BPA and alternatives across all toys.

**Results:**

Among individual toy archetypes, the teething ring, cowboy suit, and doll resulted in the highest daily exposure dose, with the exposure dose generally decreasing with age. Direct dermal contact, mouthing, and dust ingestion were the primary exposure pathways. The estimated dose varied with the toy’s material, the chemical’s properties and initial mass fraction, and children’s use patterns. For aggregate exposure from multiple toys used by 3–6-year-olds, bisphenol F resulted in the highest daily exposure (2.6 µg/kg/d), while bisphenol AP had the lowest (0.14 µg/kg/d). Aggregate exposure to BPA and alternatives was dominated by different pathways depending on chemical properties. We also estimated the aggregate exposure mass of BPA during early childhood (6 months to <12 yrs) to be 13.4 mg.

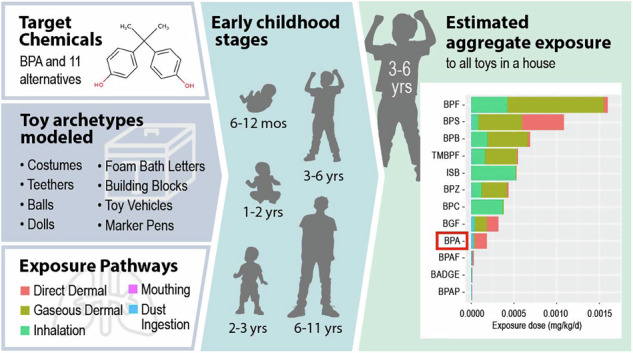

**Impact Statement:**

This study offers insights into children’s exposure to BPA and its alternatives in toys, filling in the knowledge gaps on exposures to these chemicals. USEtox modeling identifies dominant exposure pathways and evaluates aggregate exposure from the use of different toy types, demonstrating the ability of USEtox to quantify exposures to both well-known chemicals as well as new alternatives. The research underscores the variability in exposure doses based on toy material, chemical properties, and children’s behavior, providing valuable science-based and quantified data to guide safer product design. Policymakers can leverage these findings to prioritize products for regulation and protect public health.

## Introduction

Bisphenol A (BPA) is a monomer chemical used primarily in the production of polycarbonate plastics, where it functions as a key building block that provides desirable properties, such as durability, high impact strength, and heat resistance [1]. BPA has historically been used in various consumer products including water bottles, tableware, epoxy resin coating of canned foods, dental sealants, and thermal receipts [2, 3]. Humans are exposed to BPA through their diet, inhalation, non-dietary ingestion of household dust, and dermal exposure, with diet as the primary source of exposure for most people [2–4]. BPA is a known endocrine disruptor and recent epidemiological studies increasingly link BPA exposure in humans to adverse health outcomes including reproductive and developmental effects, metabolic diseases, and other health effects [2].

Children are particularly vulnerable from exposures to endocrine disruptors like BPA, and may experience long-lasting adverse health impacts after exposure due to their developing organs, higher metabolic rates, lower body weights, and unique behavior patterns [5–7]. Despite the evidence of its endocrine disrupting effects, BPA is still widely used in children’s products, including toys. Currently, the United States (U.S.) based Interstate Chemicals Clearinghouse (IC2) High Priority Chemicals Data System, an online database that includes chemicals of concern in children’s products reported in Oregon and Washington, contains 62 records of BPA in toys [8]. Only the European Union regulates BPA in toys under the Toy Safety Directive 2009/48/EC, which defines migration limits of BPA from toys intended for use by children under 36 months or toys intended to be placed in the mouth. In the U.S., there are no federal or state regulations that restrict the use of BPA in toys, although the U.S. Food and Drug Administration (FDA) has prohibited the use of BPA-based materials in baby bottles, sippy cups and infant formula packaging and California will prohibit the use of bisphenols in juvenile (up to 12 years) feeding, sucking, or teething products in 2026 [9]. Several U.S. states have reporting requirements for BPA in children’s products [10–12] since children who use these products are exposed to BPA daily and studies indicate BPA is one of the top chemicals of health concern in children’s products [13].

Non-dietary sources have been identified as potentially important contributors to total daily exposure to BPA in young children [14]. Previous studies, which have focused on toys as a non-dietary exposure source of BPA, measured the BPA migration rates from various toys and estimated the resulting children’s exposure via mouthing [5, 15–20]. These studies only consider a single exposure pathway (i.e., mouthing), while ignoring other pathways that may be relevant for toys, such as dermal contact, dust ingestion, and inhalation [21–23]. As a result, these studies may significantly underestimate children’s aggregate BPA exposure from toys. In addition, these studies generally used random toy samples collected from the local market and took average BPA migration rates across all samples, without systematically studying and comparing the various toy categories. As a result, they offered limited insight on the types of toys and exposure pathways contributing most significantly to BPA exposure.

Only a few studies have assessed children’s aggregate exposure to BPA from non-dietary sources. Wilson et al. [24] measured the aggregate exposures of nine preschool children using samples of indoor and outdoor air, play area soil, floor dust, duplicate diets, hand surface wipes, and urine. They estimated the aggregate daily dose of BPA to range from 18 to 71 ng/kg/d, which included inhalation, dietary ingestion, and non-dietary ingestion pathways [24]. However, this study did not estimate aggregate exposure from toys, as it did not differentiate between exposure from toys and from other non-dietary sources. This study focused on children aged 2–5 years but did not include children under 2 who have increased mouthing behaviors and are expected to have higher non-dietary exposures. It was also a cross-sectional study for a 48-h sampling period, which did not provide a comprehensive picture of children’s aggregate exposure throughout early childhood.

Due to concerns about the health effects of BPA, many “BPA-free” toy products using alternatives have emerged on the market. These fall into two categories: chemical substitutes and material substitutes. Chemical substitutes include petroleum-based chemicals (e.g., bisphenol S (BPS), bisphenol F (BPF), bisphenol A diglycidyl ether (BADGE)) and biomass-based chemicals (e.g., bisguaiacol F (BGF), isosorbide (ISB)) [25, 26]. Material substitutes include replacing polycarbonate and epoxy resin in toys with different types of plastics or natural materials. Each approach has tradeoffs regarding their application, complexity of production process and chemical composition, hazard profile, and data availability. Despite the availability of BPA-free alternatives on the market, their exposure and safety in toys remain largely unassessed. There is emerging evidence showing that BPA-free products containing BPS or BPF may be regrettable substitutes, as both display reproductive toxicity and endocrine disrupting properties [26, 27].

Significant knowledge gaps make it challenging to characterize chemical exposures from toys and, thus, to regulate children’s toys containing BPA or its alternatives to protect children’s health. These include gaps in understanding (1) the landscape of children’s toys containing BPA, (2) levels of BPA in children’s toys, (3) predominant exposure pathways and products leading to high exposure, and (4) the exposure and safety of potential alternatives to BPA. The overall goal of the present study is to apply modeling tools to systematically evaluate and compare young children’s exposure to BPA and potential alternatives in toys. For this purpose, we selected the USEtox model, which is endorsed by UNEP-SETAC (United Nations Environment Program—Society of Environmental Toxicology and Chemistry) Life Cycle Initiative for characterizing human exposure and health impacts of chemicals (https://usetox.org/). USEtox is a multimedia, multi-pathway model, and its latest version USEtox 3 can model the various release mechanisms and exposure pathways associated with chemicals in consumer products [28], including their release to air and subsequent inhalation exposure, release to dust and subsequent dust ingestion exposure, mouthing exposure due to migration to saliva, dermal contact exposure, etc. Compared to other consumer exposure estimation models such as ConsExpo [29], SHEDS-HT [30], CEM [31], and RAIDAR-ICE [32], USEtox 3 reflects the scientific consensus of international experts, and offers a mechanistic way to account for the mass balance and inter-dependence among multiple exposure pathways. USEtox 3 provides dynamic simulation for chemicals in consumer products. Specifically, it can model the dynamic emission, migration, and indoor sorption of chemicals embedded in solid objects (e.g., toys) which are not covered by the other exposure models. Additionally, USEtox 3 is able to perform product-specific assessments, and is easily customized to capture product characteristics and use patterns. The framework and sub-models in USEtox 3 have been published in several peer-reviewed journal articles [28, 33–38], and have been successfully applied to chemicals in plastic toys, building materials, interior paints, personal care, and household products in previous studies [13, 37, 39–41]. Thus, applying USEtox 3 enables a comprehensive assessment of multi-pathway exposures to BPA and alternatives in toys.

For alternatives, we consider chemical substitutions only, since switching to different materials needs to be evaluated by life cycle assessments which are out of scope of the present study. The specific aims of the present study include:

1. Define representative toy archetypes (Section “Toy archetypes”) that contain BPA.
2. Assess young children’s (below age of 12 years) exposure levels of BPA in individual toy archetypes and identify the dominant exposure pathways; examine the effect of child’s age, contact level, and material type of toys on the exposure.
3. Compare the exposure dose and pathways for individual toy archetypes between BPA and potential alternatives; investigate the key parameters that determine the exposure levels and dominant pathways.
4. Estimate the aggregate exposure to BPA and alternatives in toys for children aged 3–6 years; compare the aggregate exposure dose to toxicity data to evaluate health risk.
5. Estimate the aggregate exposure mass of BPA in toys for a child throughout early childhood (i.e., aggregating BPA exposure over multiple years for a child).

## Methods

### Overall assessment framework

The present study applies USEtox 3, which is a UNEP-SETAC consensus exposure and toxicity model for chemicals emitted to the environment and chemicals in consumer products (https:// usetox.org/). USEtox 3 is a mass-balance based multimedia model that builds on the Product Intake Fraction (PiF) framework [33, 42], and consistently couples chemical fate and human exposure in the far-field and near-field environments. The PiF is a metric used to quantify and compare exposure to chemicals in consumer products, which is defined as the chemical mass taken in by the user over a given exposure period per unit mass of a chemical originally in a product [42]. Far-field refers to environments that are distant from the use of a considered product, including ambient air, freshwater, soil, sediment, and biota, while near-field refers to environments within the vicinity of product users, including indoor air, objects/products and their surfaces [33]. Using the model involves the following major steps:

1. USEtox 3 first quantifies the initial mass of a chemical used in a toy, by multiplying the mass of a toy and the mass fraction of the chemical in the toy. The chemical mass fractions are typically obtained from product composition data or product testing studies, as detailed in Section “Modeling procedures”.
2. USEtox 3 estimates exposure pathway-specific PiFs for the child (user) using the toy. PiFs are estimated using a series of mechanistic, mass-balanced models, which are detailed in Section “Emission and exposure models” and previous publications [13, 28].
  The exposure pathways considered in the present study include inhalation, direct dermal contact, dermal gaseous uptake, dust ingestion, and ingestion through direct mouthing of toys.
3. The PiF is then multiplied by the initial chemical mass in the toy to calculate the child’s intake, in mg, which can be divided by the child’s body weight and the exposure duration to obtain the daily exposure dose, expressed in mg/kg/d. The exposure duration is determined by the toy’s usage, while the child’s body weight is built-in in USEtox 3 and is obtained from the U.S. Environmental Protection Agency’s (EPA) Exposure Factors Handbook (EFH) [43].
4. Exposure doses can then be aggregated across exposure pathways by exposure routes such as inhalation, ingestion, and dermal. For example, doses via direct dermal and gaseous dermal pathways are summed to obtain total dermal exposure, while dust ingestion and mouthing pathways are summed to obtain total non-dietary ingestion exposure.

### Target chemicals

The chemicals in the present study include BPA and 11 potential alternatives (Table 1). These alternatives were selected based on structural similarity to BPA, reported functional use in polymers and resins, and relevance to toy applications. The selected chemicals represent three major categories: commonly used BPA analogs (e.g., BPS, BPF), which are widely used in industrial applications and have been identified as regrettable substitute due to similar hazard profiles [26, 27]; claimed safer substitutes, such as TMBPF and BADGE, which have been proposed in the literature or regulatory evaluations as lower hazard replacements [25, 26]; and biomass-based chemicals derived from plant-based feedstocks, including BGF and isosorbide, which have attracted attention as more sustainable alternatives [44–46]. The full selection process is detailed in Appendix A, Section A1. To identify the physiochemical properties of these chemicals required as inputs for USEtox modeling, priority was given to peer-reviewed experimental data, followed by OPERA estimations [47], then EPISuite estimations [48]. The detailed substance input data and data sources for these 12 chemicals are provided in Appendix B, sheet “Substance inputs”.

**Table 1:** Upper part: selected target chemicals with their physiochemical properties, including molecular weight (MW), octanol-water partition coefficient (K_ow_), octanol-air partition coefficient (K_oa_), and Henry’s Law Constant at 25 °C (K_H_25C); Lower part: selected toy archetypes, default age group of child user, and the two possible material types.

CAS	Chemical	MW^†^	K_ow_	K_oa_^#^	K_H_25C
		(g/mol)	(–)	(–)	(Pa.m^3^/mol)
80-05-7	Bisphenol A (BPA)	228.3	2.09E + 03 *	8.98E + 10	5.76E–05 *
80-09-1	Bisphenol S (BPS)	250.3	1.73E-01 ^†^	4.42E + 08	7.92E–07 ^†^
620-92-8	Bisphenol F (BPF)	200.2	8.66E + 01 ^†^	2.49E + 07	8.63E–03 ^†^
843-55-0	Bisphenol Z (BPZ)	268.4	6.60E + 03 ^†^	2.26E + 08	7.23E–02 ^†^
14868-03-2	Bisphenol C (BPC)	281.1	3.28E + 03 ^†^	1.44E + 06	5.62E + 00 ^†^
1478-61-1	Bisphenol AF (BPAF)	336.2	2.95E + 04 ^§^	1.25E + 12	5.76E–05 ^§^
1571-75-1	Bisphenol AP (BPAP)	290.4	7.24E + 04 ^§^	3.18E + 15	5.64E–08 ^§^
77-40-7	Bisphenol B (BPB)	242.3	3.60E + 03 ^†^	1.26E + 08	7.06E–02 ^†^
1675-54-3	Bisphenol A diglycidyl ether (BADGE)	340.4	6.92E + 03 ^§^	3.85E + 12	4.45E–06 ^§^
5384-21-4	Tetramethyl bisphenol F (TMBPF)	256.3	5.59E + 03 ^†^	1.50E + 08	9.20E–02 ^†^
3888-22-0	Bisguaiacol-F (BGF)	260.3	3.12E + 02 ^†^	2.44E + 09	3.17E–04 ^†^
652-67-5	Isosorbide (ISB)	146.1	7.59E-02 ^†^	2.92E + 03	6.44E–02 ^†^
*Toy archetype characteristics*					
**Toy archetype**	**Default age group**	**1st material**		**2nd material**	
Costume	3 to <6 years	Polyethylene terephthalate (PET)		Polyamide (PA)	
Teething ring	6 to <12 months	Silicone rubber		Flexible polyvinyl chloride (PVC)	
Ball	3 to <6 years	Polyurethane (PU)		Flexible polyvinyl chloride (PVC)	
Doll	2 to <3 years	Flexible polyvinyl chloride (PVC)		Acrylonitrile butadiene styrene (ABS)	
Foam bath letters	2 to <3 years	Ethylene vinyl acetate (EVA)		Flexible polyvinyl chloride (PVC)	
Building blocks	3 to <6 years	Acrylonitrile butadiene styrene (ABS)		Polycarbonate (PC)	
Toy vehicle	6 to <11 years	Acrylonitrile butadiene styrene (ABS)		Polystyrene (PS)	
Marker pen	6 to <11 years	Polypropylene (PP)		Polycarbonate (PC)	

^*^ Peer-reviewed experimental data.

^†^ OPERA prediction.

^§^ EPISuite prediction.

^#^ ${K}_{{oa}}$ is calculated from ${K}_{{ow}}$ and ${K}_{H}25C$ by: ${K}_{{oa}}=\frac{{K}_{{ow}}}{{K}_{H}25C/(R\cdot T)}$, where $R$ = 8.314 J/(mol·K), $T$ = 298.15 K.

### Toy archetypes

We developed toy archetypes that cover a wide diversity of types of toys that children would play with. To explore plausible/realistic exposure scenarios, we compiled information on the function of toys and types of play (e.g., pretend and role play, building, media, sports, exploratory and practice, bath) from the guidelines set by the U.S. Consumer Product Safety Commission (CPSC) [49], along with children’s preferences and abilities. The types of play and representative toys were then matched with age groups (i.e., 6 months to 11 years old) outlined in the EPA EFH [43] for non-dietary exposure factors (i.e., object to mouth frequency). For the compiled list of toys, we conducted a search in the IC2 High Priority Chemicals Data System, to identify those containing BPA. The combination of age groups and toys representing pertinent type of play for the age resulted in 525 exposure scenarios for BPA in toys. These were further analyzed, resulting in 8 representative toy products, so called “Toy archetypes” (Table 1). The toy archetypes were selected based on the toy function, intended age group of children, availability of BPA concentration data, and availability of toy characteristics and usage data. Detailed rationale is provided in Appendix A, Section A2.

Material type is a key input for USEtox 3 because it is used in estimating chemical emissions and human exposure. Table 1 presents the two most commonly used material types for each specific toy archetype, such as costume and teething ring. For the toy archetypes that are commonly made of a single material type (e.g., building blocks and marker pen), we assumed the second material to be polycarbonate (PC) since BPA is a monomer used to produce PC, so it is mostly likely found in PC [1]. For modeling purposes, each toy archetype was assigned a default age group of child users (Table 1), per guidelines of the CPSC [49]. As additional plausible scenarios, we modeled exposure for children playing with toys outside the intended target age range (as described in Section “Aggregate exposure to BPA during early childhood”). Specific exposure factors were considered for different toy archetypes when data were available, such as dermal contact area and mouthing frequency. However, uniform dermal contact frequency and hand-to-mouth contact frequency were used across toy archetypes for a specific age group due to lack of toy-specific data. The detailed product inputs for USEtox 3 modeling for each toy archetype are presented in Appendix B, sheet “Product inputs”.

### Modeling procedures

Children’s exposures to BPA and alternatives in toys were modeled using the following 4-step approach:

1. To assess exposures to BPA and alternatives in individual toys, we first applied the default age group for each toy archetype, studying the effect of contact level and the toy’s material type on exposure, identifying the dominant exposure pathway(s), and investigating the key parameters that determine the exposure levels and dominant pathways.
2. Next, we explored how exposures to BPA in toys vary with children’s age.
3. We then estimated the aggregate exposure to BPA and alternatives in toys for specific age groups and combined aggregate exposure doses with toxicity data to evaluate health risk.
4. Finally, we estimated the aggregate exposure to BPA in toys over the course of early childhood to identify the time periods that contribute most to the aggregate exposure, as well as the dominant exposure pathway(s).

The detailed modeling setup is provided in Appendix A, Section A3. More information on model description and model inputs is presented in Sections “Emission and exposure models” and “Mass fractions of BPA and alternatives in toys”.

### Emission and exposure models

We used the built-in modeling set “Model_Article_Interior” in USEtox 3 to predict children’s exposures to BPA and its alternatives in toys, as it is well-suited and has previously been used to assess exposures to chemicals in toys [28]. This model integrates several sub-models that predict chemical emissions from solid materials, chemical migration from solid materials to saliva, as well as exposures via inhalation, dermal gaseous uptake, dermal contact, dust ingestion, and mouthing ingestion. These sub-models are described in more detail below.

#### Chemical emissions from solid materials

USEtox 3 integrates “the combined D- and K-limited model with sorption” [34], which models chemical emissions as the combination of chemical diffusion inside the solid material and chemical partition between the solid material and air at material surface. This model was originally developed for building materials [34, 39] and has been successfully applied to toys [13].

#### Inhalation and dermal gaseous exposures

Once chemicals are emitted to indoor air, this model calculates the chemical concentration in indoor air, which is multiplied by the inhalation rate to calculate inhalation exposure, and multiplied by a total gaseous-skin permeation coefficient and skin surface area to determine dermal gaseous exposure [13].

#### Dermal contact and dust ingestion exposures

These models are directly related to the model predicting chemical emissions from solid materials. The emission model also predicts the time-varying chemical concentration at the material surface, which is then combined with the chemical material-water partition coefficient, the skin permeation coefficient via aqueous solution, and the dermal contact frequency to calculate dermal contact exposures [13]. Similarly, the chemical concentration in dust is assumed to be in equilibrium with the chemical concentration at the material surface; a material-dust partition coefficient is combined with the dust ingestion rate to calculate chemical exposure via dust ingestion [13].

#### Chemical migration from solid materials to saliva for mouthing exposure

To predict mouthing exposures we adapted a model predicting chemical migration from food contact materials (FCMs), considering the toy as the FCM and the saliva as the food medium. We considered the mouthing duration and frequency, mouthing contact area, temperature inside the mouth, and the saliva’s volume and ethanol equivalency when adapting the FCM model. This model has been successfully applied to estimate mouthing exposure to chemicals in children’s products, and it has been evaluated against experimental toy-to-saliva migration rates for various chemical groups and toy materials [37]. Chemical migration to saliva and emission to indoor air were considered as two competing processes.

#### Contact level

We considered the variability in the child’s contact level with the toys for dermal contact, dust ingestion, and mouthing exposures. We used the “average” and “high-end” contact levels built into USEtox 3, each corresponding to different values of dermal contact frequency, hand-to-mouth contact frequency, and mouthing frequency for broad categories of “Toy”, “Toys meant for mouthing”, or “Other toys”. The values of these parameters were taken from the EPA EFH [43] and ConsExpo Children’s Toys Factsheet [50].

All model runs were performed in USEtox 3 using the version “USEtox_3.0beta7d.xlsm”. The indoor environment used for modeling was “OECD countries average 2 + 1 children household” with a volume of 236 m^3^ and an air exchange rate of 0.79 h^-1^. The play scenarios and child characteristics are specific to each toy archetype and age group, with details provided in Appendix B, sheets “Product inputs” and “Age groups”. The chemical-product specific inputs, including the chemical internal diffusion coefficient in the (toy) product and its product-air partition coefficient, were estimated using quantitative structure-properties relationships (QSPRs) built into USEtox 3 [51, 52]. The diffusion coefficient is predicted from the chemical’s molecular weight, the material type of the product, and the temperature [52], while the product-air partition coefficient is predicted from the chemical’s octanol-air partition coefficient, the material type of the product, and temperature [51].

### Mass fractions of BPA and alternatives in toys

Data on the range of mass fractions for BPA and alternatives in each toy archetype are essential inputs to accurately estimate the BPA exposure levels. In this study, we focus on BPA and alternatives that are used intentionally as functional ingredients in toys. Since the availability of mass fraction data is extremely limited, we used a point estimate of 300 ppm as the mass fraction of BPA and alternatives across all 8 toy archetypes based on the IC2 database. We have also constructed possible mass fraction ranges for BPA and alternatives in toys and conducted a sensitivity analysis. The details are provided in Appendix A, Section A5.

## Results

### Children’s exposure to BPA in toys

This section focuses on estimated exposures to BPA, starting from a product perspective and assessing the total exposure dose for individual toys across multiple exposure pathways. Next, we examine the variation in BPA exposure across age groups for individual toys. Finally, we take a receptor perspective and consider the aggregate exposure across all toys that a child has during early childhood, from age 0 through 11.

#### Exposure to BPA in individual toys

**Fig. 1:**
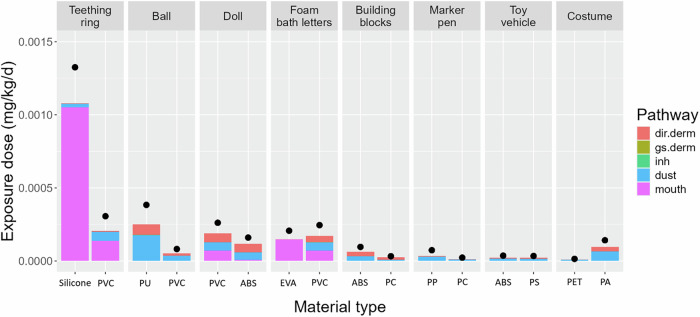
Comparison of the effect of material type on BPA exposure dose for eight toy archetypes, broken down by route. Each toy archetype was assumed to be played with by the default age group. For each toy archetype, the left part represents the 1st (default) material type, and the right part represents the 2nd material type; the bars represent the exposure doses with an average contact level, and the black circles represent the exposure doses with a high-end contact level. PVC polyvinyl chloride, PU polyurethane, ABS acrylonitrile butadiene styrene, EVA ethylene vinyl acetate, PC polycarbonate, PP polypropylene, PS polystyrene, PET polyethylene terephthalate, PA polyamide.

Figure 1 presents the estimated BPA exposure dose summing all exposure pathways for each toy archetype and its default age group, comparing average and high-end contact levels, and comparing the two plausible material types. With the average contact level and default material type, the BPA exposure dose is highest for the teething ring, where mouthing is the dominant pathway, followed by the bouncy ball, where dust ingestion is the dominant exposure pathway. Figure 1 also demonstrates that exposure doses are highly sensitive to the toy material type. For example, the BPA exposure dose for the teething ring with average contact level is the highest among all toys (1.08 × 10^–3 ^mg/kg/d) with silicone rubber as the material but decreases by a factor of 5 (2.07 × 10^–4 ^mg/kg/d) with PVC as the material. Similarly, BPA exposure estimates for the bouncy ball and costume vary by factors of 5 and 9, respectively, depending on material types. Thus, toy material type can be a major source of uncertainty and can significantly affect the estimated exposure, as discussed in Section “Uncertainties on key parameters”.

The dominant exposure pathway is also strongly influenced by the material type. For example, when the material type is flexible PVC, mouthing, direct dermal contact and dust ingestion contribute equally to BPA exposure from foam bath letters. In contrast, when EVA is the material, exposure is dominated by mouthing (Fig. 1).

The dominant exposure pathway is also dependent on the toy archetype and its related product use pattern. Exposures to BPA in teething rings and foam bath letters are dominated by mouthing, due to the young age of the default users (6 to <12 months for teething ring and 2 to <3 years for foam bath letters). Mouthing, direct dermal contact, and dust ingestion contribute equally to BPA exposure for the doll, while dust ingestion and direct dermal contact are the two most important exposure pathways for the bouncy ball, building blocks, marker pen, toy vehicle and costume.

We estimated BPA exposures for children in different age groups for the three toy archetypes that would be played with by all of them: the doll, building blocks, and toy vehicle. The results are presented in Appendix A, Section A4. While the exposure dose per unit body weight is significantly lower for older children relative to their younger counterparts, due to their greater body weight and reduced mouthing behavior, the ranking of the contribution of the three toy archetypes remains the same across all ages: doll > building blocks > toy vehicle.

#### Aggregate exposure to BPA during early childhood

**Fig. 2:**
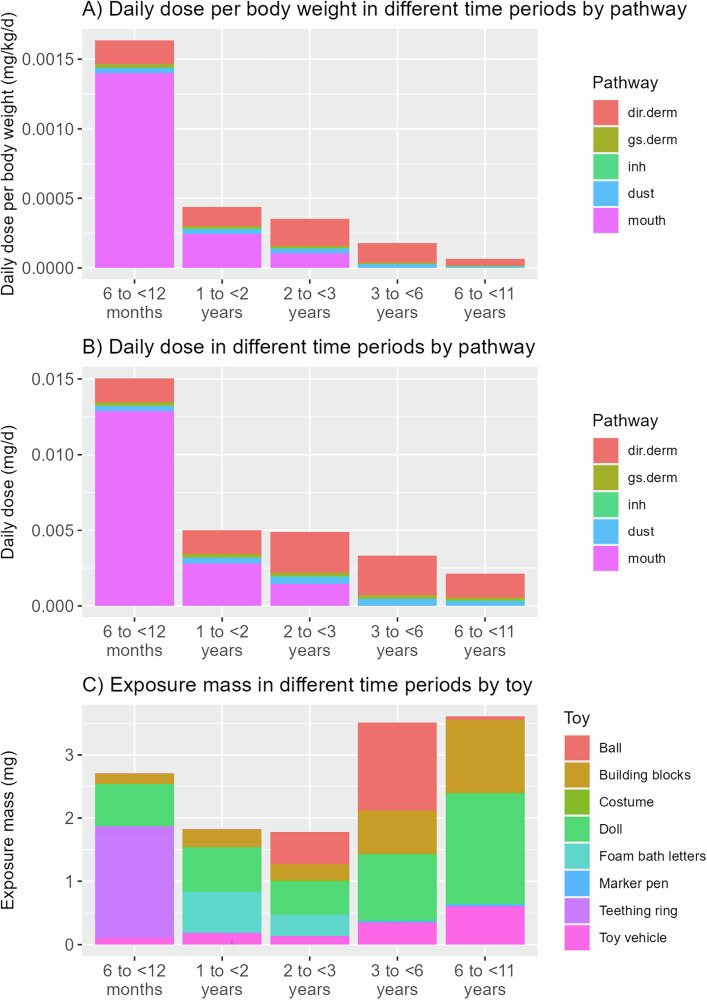
Aggregate exposure to BPA from all toys for a child throughout early childhood (i.e., 6 months to 11 years). The figure shows (**A**) the daily exposure dose per body weight (mg/kg/d) for different age ranges by exposure pathway, (**B)** the daily exposure dose (mg/d) for different age ranges by exposure pathway, and (**C)** the exposure mass (mg) for different age ranges by toy archetype.

Figure 2 presents the estimated aggregate exposure of a child to BPA, from all toys, between the ages of 6 months and 11 years. A child’s highest daily exposure dose per body weight (mg/kg/d) clearly decreases with age, with the highest occurring at the age of 6 to <12 months and the lowest occurring at the age of 6 to <11 years (Fig. 2A). This is mainly due to reduced mouthing exposure and increased body weight as age increases. Multiplying the daily dose per body weight (mg/kg/d) by the child’s body weight results in the daily dose per person (mg/d) (Fig. 2B). The daily dose per person generally follows the trend of the daily dose per body weight, but the difference between time periods becomes smaller due to the increasing body weight over time.

The exposure mass (mg) was further calculated by multiplying the daily dose per person by the duration of each time period (Fig. 2C). The exposure mass displays a distinct trend from the daily dose, with the highest exposure mass occurring at the age of 6–<11 years, mainly due to the prolonged duration of toy use (5 years) compared to other time periods. In contrast, the lowest exposure mass occurs at the age of 2–<3 years due to the relatively low daily dose and short duration (1 year).

Throughout early childhood, BPA exposure varies by toy and exposure pathway across different time periods. While mouthing exposure from teething rings, dolls and foam bath letters dominate the exposure when the child is an infant, direct dermal contact with dolls, building blocks and balls plays a more important role when the child grows into a toddler and finally a school-aged kid. Summing across all age groups, the aggregate exposure mass of BPA for a child from 6 months to 11 years is estimated to be 13.4 mg. This estimate may vary depending on the toys’ material types, the BPA mass fraction in the toys, the child’s use pattern and characteristics of the indoor environment, as further discussed in Section “Uncertainties on key parameters”.

### Comparison between BPA and alternatives

This section compares toy-related exposures to BPA and 11 chemical alternatives (listed in Table 1). We first analyze individual toy products as drivers for exposure. We then analyze children as receptors to the aggregated exposures from different toys and, finally, estimate the related health risks.

#### Exposure to BPA and alternatives in individual toys

**Fig. 3:**
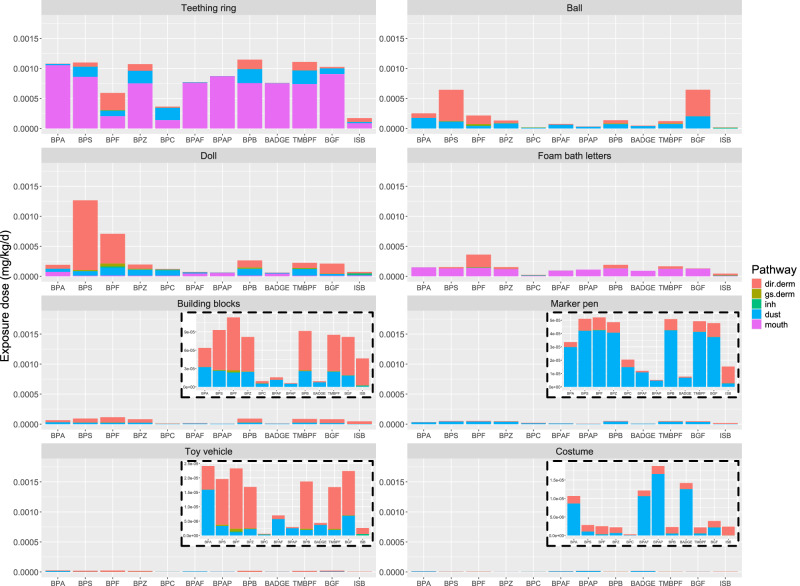
Exposure dose for BPA and the 11 alternative chemicals for each toy archetype. The exposure doses were estimated with the default age group, default material type and average contact level, and are presented by exposure pathway.

Figure 3 compares the estimated exposure dose, by pathway, between BPA and 11 alternatives for each toy archetype. For the teething ring, the highest exposure dose is for BPB, closely followed by BPA, BPS, BPZ, TMBPF, and BGF. For the ball and doll, the highest exposure dose is for BPS, followed by BPF, or BGF, while for foam bath letters, the highest exposure dose is for BPF. The exposure doses from building blocks, marker pens, toy vehicles and costume are much lower than those from other toys. ISB, BPC, BPAP, BPAF and BADGE result in substantially lower exposure doses than BPA in all toys except the costume, for which BPAF, BPAP, and BADGE result in higher doses (however, very low exposures are estimated for all target chemicals in the costume).

The dominant exposure pathway also differs by toy archetype and chemical. For the toys used by younger children—the teething ring and foam letters—mouthing is the dominant pathway for all chemicals. Dust ingestion is dominant for BPAF, BPAP and BADGE in all other toys except the PVC doll, due to their high K_oa_ and thus K_ma_. Interestingly, mouthing is the dominant exposure pathway for BPAF, BPAP, and BADGE in the PVC doll since the concentration in dust of these high K_oa_ chemicals tends to be lower on the surface of PVC than on that of other materials; this results in a lower contribution from the dust ingestion pathway and a correspondingly higher contribution from the mouthing pathway. Dust ingestion is also the dominant pathway, at low exposure levels, for most chemicals in the marker pen and costume. Inhalation is usually negligible except for dominant low-level exposures to BPC and ISB in the ball and doll, due to their low K_ma_. Direct dermal exposure is dominant for all other chemical-toy combinations for the ball, doll, building blocks, toy vehicle, marker pen, and costume.

In summary, BPS, BPF, and BGF generally have higher exposures across individual toys, and the exposures are mainly contributed by direct dermal contact and mouthing.

#### Aggregate exposure to BPA and alternatives for a specific age group

**Fig. 4:**
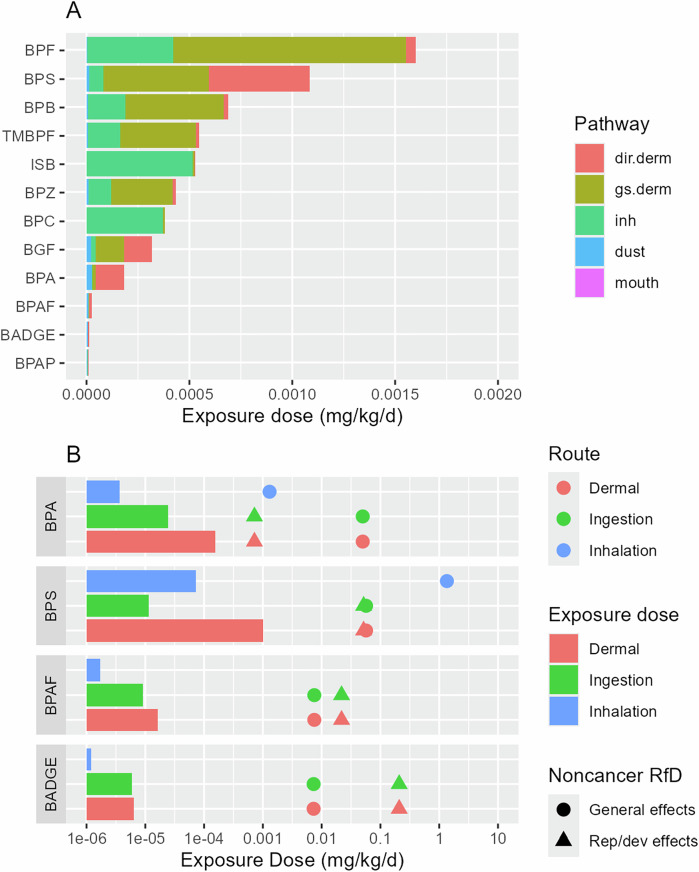
Aggregate exposure to BPA and the 11 alternatives for children 3- <6 years and the associated health risks. The figure shows (**A**) the aggregate exposure from all 54 kg of toys found in the home, by exposure pathway, and (**B)** the comparison between aggregate exposure and reference dose (RfD) for BPA, BPS, BPAF, and BADGE.

Figure 4A presents the estimated aggregate exposure from all 54 kg of toys that can be found in the homes of children 3–<6 years in the U.S. The contribution from each toy archetype is detailed in Appendix A, Section A5. The age group of 3–<6 years was selected since all 8 toy archetypes except the teething ring would be played with by this age group, the most among all age groups. For BPA, the aggregate exposure is dominated by direct dermal contact followed by dust ingestion, with the bouncy ball and doll contributing the most (Fig. A2). This indicates a child’s exposure to BPA from toys mainly comes from direct contact during play. BPAF, BPAP, and BADGE display aggregate exposure patterns similar to those of BPA, with exposure dominated by direct dermal contact and dust ingestion during play. The balls contribute most to aggregate exposure to BPAF and BADGE, and the dolls to aggregate exposure to BPAP.

Direct dermal contact and gaseous dermal uptake (Fig. 4A) contribute equally to aggregate exposure to BPS and BGF, with the dolls contributing the most (Fig. A2). Thus, for these two chemicals, a child’s exposure results from both direct contact when playing with the toys and the air emissions from all the toys stored in the house.

For the remaining chemicals BPF, BPZ, BPC, BPB, TMBPF, and ISB, the aggregate exposure is dominated by gaseous dermal uptake and inhalation pathways (Fig. 4A), with dolls and bouncy balls contributing the most (Fig. A2). In particular, for BPC and ISB which have the lowest logK_oa_ values among all target chemicals, the aggregate exposure is dominated by inhalation. For these chemicals, the primary source of a child’s exposure is all the toys stored in the house, instead of the toys being directly played with.

Among BPA and the 11 alternatives, BPF has the highest aggregate exposure dose in children 3 to <6 years (0.0016 mg/kg/d), followed by BPS (0.0011 mg/kg/d). BPAP (8.63 × 10^–6 ^mg/kg/d), BADGE (1.36 × 10^–5 ^mg/kg/d) and BPAF (2.71 × 10^–5 ^mg/kg/d) have the lowest aggregate exposure doses (Fig. 4A). Interestingly, the aggregate exposure dose for BPA (1.82 × 10^–4 ^mg/kg/d) is the fourth lowest among the 12 target chemicals.

Figure 4B compares the aggregate exposure for children 3 to <6 years to toxicity benchmarks to assess the potential health risk. The reference doses (RfDs) for noncancer effects are presented, as no cancer toxicity data are available for any of the target chemicals. Two RfDs are presented for each chemical for general noncancer effects (non-reproductive or developmental) and for reproductive or developmental (rep/dev) effects [53, 54]. All RfDs are probabilistic values except for BPA’s ingestion RfD for general noncancer effects, which is a regulatory value (0.05 mg/kg/d) [53, 54]. The probabilistic RfDs were derived statistically from experimental animal data and reflected the lowest 95% confidence interval on the 1% incidence effect dose [53, 54]. RfDs are only available for 4 of the 12 chemicals: BPA, BPS, BPAF, and BADGE. As shown in Fig. 4B, none of the estimated aggregate exposures to these four chemicals exceeds the corresponding RfD. However, dermal exposure to BPA is only a factor of 4 lower than the RfD for rep/dev effects. We also conducted a sensitivity analysis by varying the chemical mass fraction in toys (Appendix A, Section A5.4). The exposure estimates change linearly with the mass fraction, and the upper estimate of dermal exposure to BPA exceeds the rep/dev RfD. It is important to note that this sensitivity analysis does not reflect the full uncertainties in the exposure estimates.

Although this comparison considers route-to-route extrapolation, it highlights the potential concern given that BPA is a known endocrine disruptor with toxic effects on reproduction and development. Although the aggregate exposure of BPA for children aged 3 to <6 years generally does not pose a significant risk, there is concern for children younger than 3 years because of their higher aggregate exposure to BPA from toys, or if the BPA mass fraction in toys exceeds 1,000 ppm.

## Discussion

Our results highlight several important findings on children’s exposure to BPA and its alternatives in toys. Among single toys, the teething ring, bouncy ball, and doll result in the highest daily exposure doses in the main users, primarily through dermal contact, mouthing, and dust ingestion. Estimated exposure doses are highly dependent on the toy’s material, the chemical’s properties, and the chemical’s initial mass fraction in the toy. When considering the aggregate exposure from all toys that a 3–6 -year-old child possesses, substituting BPF or BPS for BPA would result in the highest daily aggregate exposure doses, while use of BPAP, BPAF and BADGE would result in the lowest. Notably, substituting any of eight BPA alternatives for BPA would result in higher aggregate doses, with the dominant exposure pathways varying by chemical. In this section, we discuss the key factors driving these observed trends of exposure.

### Key chemical properties determining exposure

Children’s exposures to BPA and its alternatives in toys are primarily related to their emissions from toys to indoor air. Our findings showed that BPA and alternatives exhibited variable emission characteristics. In the present study, such emissions are estimated in USEtox 3 by multiplying the chemical’s initial mass in toys (e.g., 0.15 g of BPA) and the mass fraction emitted (e.g., 10% of BPA would be emitted to air), where the mass fraction emitted is estimated by “the combined D- and K-limited model with sorption” in USEtox 3 (Section “Emission and exposure models”). The two key input parameters for this model are the chemical’s diffusion coefficient inside the solid material (i.e., toy) (D_m_) and the chemical’s partition coefficient between the solid material and air (K_ma_). They are determined by both the material type of the toy and the chemical’s properties in USEtox 3 (molecular weight for D_m_ and K_oa_ for K_ma_) [51, 52].

**Fig. 5:**
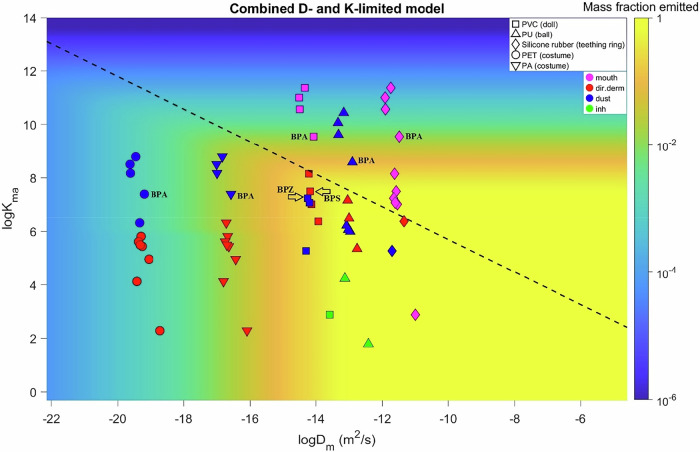
Effects of diffusion coefficient and material-air partition coefficient on the chemical emission. The heat map shows the mass fraction emitted (to indoor air) of chemicals over a 1-year duration as a function of the 10 logarithms of the diffusion coefficient (X-axis) and the material-air partition coefficient (Y-axis) for a toy with a thickness of 0.0019 m. The circle, square, triangle, and diamond symbols show the positions on the heat map of BPA and 11 alternatives in different materials. The colors of the symbols show the dominant exposure pathway (as shown in the legend) for specific chemical-material combinations. The black dashed line indicates the line separating the D-limited (below the line) and K-limited (above the line) zones. The D_m_ and K_ma_ values shown in this figure are provided in Appendix B, sheet “D and Kma”.

The heat map of Fig. 5 shows how emissions vary with D_m_ and K_ma_, highlighting the D_m_ and K_ma_ values of the 12 selected chemicals for 5 materials used in 4 toys: silicone rubber (teething ring), PU (ball), PVC (doll) as well as PET and PA (costume). Below the dashed diagonal, the fraction emitted is driven by the diffusion coefficient as D_m_ increases from 10^-22^ to 10^-13^ m^2^/s, above which 100% of the chemical is emitted within one year. Above the diagonal, it is K_ma_ that limits the emission. 100% of the chemical content is emitted with a logK_ma_ below 7, while this fraction is reduced as logK_ma_ increases up to 12.

The material type significantly affects exposure estimates, mainly due to its strong influence on the D_m_. For the same material type, D_m_ values are similar across the 12 chemicals, but they can vary by up to 10 orders of magnitude across different material types. On the other hand, K_ma_ is driven by chemical properties, as its values can vary by up to 10 orders of magnitude across chemicals for the same material type, but the variation between material types is small. Such characteristics are a result of the QSPRs used to estimate D_m_ and K_ma_ in USEtox 3 [51, 52]. Figure 5 illustrates that the two material types, PET and PA, for the same costume result in diffusion coefficients that differ by 3–4 orders of magnitude. This leads to substantial differences in chemical emissions (mass fraction emitted is around 10^–4^ for PET and around 10^–2^ for PA), thereby explaining the large exposure differences observed in Fig. 1. It is thus ideal to have material-specific diffusion coefficients or even material-specific emission/migration data to accurately assess chemical exposure.

The D_m_ and K_ma_ values do not only affect the amounts of chemicals emitted to air, but also the dominant exposure pathway(s). Figure 5 also shows the dominant exposure pathway by the color of the symbols. In the K-limited zone, which is above the dashed diagonal line, the chemical-product combinations are dominated by either dust ingestion or mouthing exposure. This is prominent for the three chemicals with the highest K_oa_ values (Table 1) and thus highest logK_ma_ (i.e., BPAF, BPAP and BADGE), where the exposure is always dominated by dust ingestion or mouthing. On the other hand, in the D-limited zone which is below the dashed diagonal line, the dominant exposure pathway is mostly direct dermal contact or dust ingestion. In particular, for the two chemicals with the lowest K_oa_ values and thus lowest logK_ma_ (i.e., BPC and ISB), the exposures from a PVC doll and a PU ball are dominated by inhalation. This is because a low K_ma_ would lead to rapid release of the chemical to the air, and high K_ma_ would limit such release but enhance the chemical sorption to dust particles or chemical migration to saliva.

Besides D_m_ and K_ma_, children’s exposure to BPA and its alternatives is also affected by other physiochemical properties, such as the chemical’s octanol-water partition coefficient (K_ow_), molecular weight (MW) and Henry’s Law constant (K_H_). For example, Fig. 5 shows two square symbols around the location with logD_m_ = -14 and logK_ma_ = 7 (indicated by two arrows). These represent BPS in the PVC doll which is dominated by direct dermal contact and BPZ in the PVC doll which is dominated by dust ingestion. BPS has similar D_m_ and K_ma_ values as BPZ, but its K_ow_ is 4 orders of magnitude lower than BPZ, leading to a high ratio between the material-water partition coefficient (K_mw_, predicted by K_ow_) and the skin permeation coefficient (K_p_aq_, predicted by MW and K_ow_), thus its higher dermal contact exposure (more details shown in Fig. 3).

### Uncertainties on key parameters

The exposure estimates for BPA and alternatives in children’s toys depend mainly on the toy’s material type, the chemical properties, the chemical’s mass fraction in the toy, and the child’s use pattern. Here we discuss the uncertainties related to these parameters.

As discussed above, the estimated BPA exposure dose is highly sensitive to the material type of the toy, which is a key input parameter for estimating the diffusion and partition coefficients in USEtox. The estimated diffusion coefficient for a given chemical can differ by 18 orders of magnitude across different material types, while K_ma_ can differ by 4 orders of magnitude. This does affect the fraction of the chemicals emitted to air as illustrated in Fig. 5.

A chemical’s properties also contribute to uncertainty in emission and exposure estimates. The logK_ma_ is a key property that varies linearly with logK_oa_, with slopes ranging from 0.67 and 0.91 depending on the toy’s materials. Thus, an uncertainty of two orders of magnitude for K_oa_ would be reflected as uncertainties between 1.33 and 1.81 orders of magnitude for K_ma_, which would further lead to uncertainties of up to 2 orders of magnitude for the emitted mass fraction, depending on the position considered on the D_m_ versus K_ma_ space of Fig. 5. In contrast, logD_m_ varies linearly with the logarithm of molecular weight (logMW), a precisely measured property which generally is not associated with uncertainty.

The indoor temperature also affects the estimated diffusion and partition coefficients in USEtox 3 and thus affects the exposure dose. However, the indoor temperature is expected to vary in a small range, especially in the U.S., so its effect is expected to be minimal. The relative humidity may also affect the chemical emission to indoor air, but this is not considered in USEtox 3.

Exposure estimates change proportionally with the chemical’s mass fraction in the toy, as illustrated in the sensitivity analysis. This study assumed a concentration of 300 ppm for BPA and its alternatives across all toys. If actual concentrations are higher—such as the 5000 ppm maximum reported in the IC2 database, this study’s estimates may underestimate exposure. However, if the BPA and alternatives are not intentionally used in toys, and trace contamination is the only source, the concentrations would be orders of magnitude lower (as found by Souza et al. [15], 0.035–3.46 ppm in 71 toy samples from Brazilian markets) and consequently, the exposure estimates would also be much lower.

A child’s use pattern of the toy, such as dermal contact frequency and mouthing frequency, would also linearly affect the estimates of dermal contact exposure, dust ingestion exposure and mouthing exposure. We used the mouthing frequency data from ConsExpo Children’s Toys factsheet [50], which assumed non zero mouthing frequency for children up to 12 months. The estimated mouthing exposure would increase if we had data indicating mouthing occurs in older age groups.

### Limitations

The first limitation of the present study is the limited amount of data quantifying concentrations of BPA and alternatives intentionally used in toys. Data on BPA alternatives is extremely limited. Our findings are based on the plausible selected concentration of 300 ppm across all alternatives and should be linearly modified for lower or higher concentrations as more data becomes available. Despite the enactment of laws in several U.S. states requiring reporting of chemical ingredients in children’s products, comprehensive data on the concentrations of chemical ingredients in toys is not publicly available. Access to such data would improve the accuracy of our aggregate exposure estimates.

Second, this study used several simplifying assumptions in estimating the aggregate exposure. We assumed all 54 kg plastic toys in a child’s house would contain BPA or alternatives and allocated the 54 kg across the 8 toy archetypes. Additionally, total dermal contact frequency and mouthing frequency were divided evenly by the number of toy archetypes a child (of a certain age group) would play with. The toy composition of the 54 kg plastic toys was assumed constant throughout the child’s entire early childhood, although we did assume the child would only play with age-appropriate toys while other toys were in storage. More refined input data on toy mass, chemical composition, and children’s use patterns would improve the accuracy of the aggregate exposure estimates.

Third, this study did not cover all toy categories. Two of the seven main toy categories defined by CPSC were excluded in this study. We excluded puzzles (made of cardboard or wood), which were not expected to contain BPA or affect the estimated BPA exposure levels. Items in the “Educational play” category (e.g., books, science sets, flashcards) may contain plastic or epoxy materials that might embed BPA or alternatives. However, these were also excluded due to the very limited data available on the characteristics, usage and BPA concentrations.

Finally, this study only considers exposure during the use stage of toys when comparing BPA and its alternatives. BPA and its alternatives are also known to leach from polycarbonate over time, potentially leading to increased BPA exposure from toys. This pathway could only be modeled if additional data becomes available and could lead to increased estimated exposures. To gain a more comprehensive understanding of the alternatives, future work should extend to long-term exposure estimates and consider a life cycle approach with other life cycle stages (production, distribution, disposal, etc.) and other impacts (global warming, ecological toxicity, acidification, eutrophication, resource use, etc.).

### Conclusion and future work

The present study is the first study to quantify children’s aggregate exposures to BPA and 11 alternative chemicals in 8 types of toys by applying USEtox 3 to various exposure scenarios. It demonstrates that estimated exposure dose is highly dependent on the toy’s material type, the chemical’s octanol-air partition coefficient, and the chemical’s initial mass fraction in the toy. This study highlights that multiple exposure pathways (not just mouthing) can contribute significantly to children’s exposure to BPA and alternatives in toys. Our findings show that multiple BPA alternatives result in higher aggregate exposure doses than BPA, indicating the need for more toxicity information on these alternatives.

Current research and regulations mainly focus on BPA migration to saliva and mouthing exposure. However, our findings show that dermal and inhalation exposures play an equal or more significant role, especially for certain BPA alternatives with distinctly different properties from those of BPA. It thus calls for more attention on exposure pathways other than mouthing, as well as toys other than those intended to be mouthed. More research is needed on the exposure to BPA alternatives; as this study shows, some of them may lead to significantly higher exposure than BPA. Future research should also prioritize addressing key data gaps, such as the concentrations of BPA and alternatives in various toys. Finally, as discussed above, a more comprehensive aggregate exposure assessment using more refined input data, taking into account the leaching of BPA and alternatives from polymers, and considering a life cycle approach would be desirable in the future.

## Supplementary information

Revised_Paper_BPA_toys_Appendix A_final

Revised_Paper_BPA_toys_Appendix B_final

## Data Availability

All data are available upon request by contacting the first author Lei Huang (lei.huang@dtsc.ca.gov).
