# Supplementary material for: USEtox modeling of children’s exposures to Bisphenol A (BPA) and alternatives in toys
Source: J Expo Sci Environ Epidemiol. 2026 Feb 12;36(3):425–37. doi: 10.1038/s41370-025-00827-6 (PMC13143805; doi:10.1038/s41370-025-00827-6)
Supplement: Supplementary file 1 — Revised_Paper_BPA_toys_Appendix A_final [file 41370_2025_827_MOESM1_ESM.docx]

**Appendix A**

**USEtox Modeling of Children’s Exposures to Bisphenol A (BPA) and Alternatives in Toys**

Lei Huang, Lynn Nakayama Wong, Xiaoying Zhou, Michelle Romero-Franco, Nathalie Pham, Hyeong-Moo Shin, Thomas McKone, Qingyu Meng and Olivier Jolliet

# A1. Selection of BPA alternatives

A1.1 Functional use of BPA

The process of selecting alternatives to Bisphenol A (BPA) in toys involves a preliminary but comprehensive evaluation of chemical functionalities, hazard profiles, and potential applications. This multi-step approach considers performance, safety, and feasibility. BPA serves as a monomer in the production of polycarbonate plastics and epoxy resins. Due to BPA’s intrinsic stability and rigid structure, the resulting polycarbonates have excellent thermal and mechanical properties, making those lightweight toys highly shatter-resistant, very strong, and durable.

A list of data sources was compiled to search for the functional use of BPA and for the identification of potential alternatives to BPA used in toys.

- **High Priority Chemicals Data System** (HPCDS; <https://hpcds.theic2.org/Search>)

The Interstate Chemicals Clearinghouse (IC2) High Priority Chemicals Data System (HPCDS) is an online platform that supports reporting information on the presence of chemicals of concern in children’s products, as required by the Oregon Toxic-Free Kids Act, the Washington Children’s Safe Products Act, and the Vermont Chemical Disclosure Program. It provides data on chemical reporting requirements under various state programs, with coverage extending beyond children's products to broader consumer product categories.

We searched the database using the keywords “bisphenol” and “toys” to obtain information on chemical-product combinations.

- **United States Environmental Protection Agency (U.S. EPA)** [**CompTox Chemicals Dashboard**](https://comptox.epa.gov/) (https://comptox.epa.gov/dashboard/)

U.S EPA compiles data from sources including the U.S. EPA’s computational toxicology research databases and public domain databases such as the National Center for Biotechnology Information’s PubChem database and U.S. EPA's ECOTox Knowledgebase, including physicochemical properties, environmental fate and transport, exposure, usage, in vivo toxicity, and in vitro bioassay into this online tool. We searched the Product category of “toys and articles intended for children's use” (e.g., stuffed toys, blankets, games, baby bottles and pacifiers, dolls), to obtain chemical-product combination as well as BPA functional use information from the database.

Other relevant databases:

- [**Pharos**](https://pharosproject.net/) (<https://pharos.habitablefuture.org>)

Habitable’s (formerly Healthy Building Network) Pharos project is a comprehensive chemical hazard database that provides GreenScreen® list translator scores, GreenScreen® Assessments (if publicly available), and regulatory information across multiple sectors including building materials and consumer products.

- [**Mintel**](https://www.mintel.com/) ([https://www.mintel.com/](https://proofpointisolation.com/browser?url=https%3A%2F%2Fwww.mintel.com%2F))

A database tracking commercial products and their ingredients across consumer goods sectors, providing detailed information about product formulations and launches worldwide.

- [**Consumer Product Information Database**](https://www.whatsinproducts.com/) (CPID; https://www.whatsinproducts.com/)

A database providing detailed information about chemical ingredients in household products, including cleaners, personal care items, toys, automotive products, and home maintenance products, along with their associated health effects and exposure information.

- **U.S. EPA Chemical and Products Database** ([CPDat](https://cadtsc.sharepoint.com/sites/spwp-ChemPET/Shared%20Documents/Collaboration%20with%20Olivier%20Jolliet/Research/BPA/BPA%20paper/CPDat); <https://www.epa.gov/chemical-research/chemical-and-products-database-cpdat>)

U.S. EPA's database that maps chemicals to their presence in consumer products, providing information about chemical concentrations, functional uses, and product categories. It combines data from multiple sources to support chemical exposure assessments.

- **U.S.** [**EPA Functional Use Database**](https://pasteur.epa.gov/uploads/495/functional_use_database.xlsx) (Fuse; <https://www.epa.gov/chemical-research/functional-use-database-fuse)>

A database documenting chemical functions across products (e.g., surfactants, solvents, preservatives), supporting informed chemical substitution in product formulations.

- [**REACH database**](https://echa.europa.eu/information-on-chemicals/registered-substances) ([https://echa.europa.eu/information-on-chemicals](https://proofpointisolation.com/browser?url=https%3A%2F%2Fecha.europa.eu%2Finformation-on-chemicals))

European Chemicals Agency's extensive database of registered chemical substances, providing information on hazard properties, classification, safe use guidelines, and regulatory requirements for chemicals in the European Union market.

We searched these databases above using terms such as “bisphenol”, “toy”, “children”, “80-05-7” etc. But the product-chemical information obtained from these databases is generally limited.

A1.2 Potential Alternatives
In this step, a preliminary list of potential alternatives was identified based on relevant functional use of BPA in toys. In the effort to identify the potential alternatives to substitute BPA in toys, we first started with 149 substances classified as bisphenol and bisphenol derivatives, which either have the generic bisphenol structure or the constitutes with the common structural features common to bisphenols [1]. For toy applications, we focused primarily on chemicals that could serve as intermediates in the manufacture of polymers and resins (particularly polycarbonates and epoxy resins) and as polymer additives. Through this functional use assessment and structural similarity comparison focusing on the presence of phenol groups and bridging elements characteristic of bisphenols, the list was narrowed down to 49 chemicals that could potentially serve similar functions as BPA in toy applications. To ensure this study remained practical and manageable, a subset of representative chemicals was selected for comparative exposure assessment. Another group of alternatives, aimed at replacing polycarbonate and epoxy resin in toys with different types of plastics or natural materials, was excluded from this study due to the suitability of the USEtox model and data availability for both composition and processes associated with these alternatives.

The potential alternative chemicals were categorized into three subgroups:

- Category 1 includes commonly used BPA analogues with similar structural and functional use, often recognized as regrettable substitutes due to similar hazard concerns from structural similarities to BPA, with simple additions or removals of side groups. Examples include bisphenol S (BPS), bisphenol F (BPF), and bisphenol AF (BPAF).
- Category 2 includes chemicals claimed to be safer substitutes, but their applications in toy products are uncertain due to limited marketplace data. Examples include BADGE and tetramethyl bisphenol F (TMBPF), which are reported to be used in epoxy and food can linings or coatings.
- Category 3 includes biomass-based chemicals produced from plant-based feedstock. Examples include bisguaiacol F (BGF) which is made from lignin from the waste in paper manufacturing process and Isosorbide (ISB), which is commercially available in the market [2–4]. These chemicals have gained recent attention because they generally have fewer hazard concerns and may have a smaller carbon footprint from a life cycle perspective.

From these three categories, eleven alternatives were ultimately selected for detailed assessment based on multiple selection criteria. First, we prioritized chemicals with sufficient data availability for USEtox modeling, including physicochemical properties and toxicological endpoints. Second, we considered regulatory relevance, selecting substances either already under regulatory scrutiny or identified as emerging alternatives in toy applications. Third, we evaluated market presence and industrial applications, focusing on chemicals with documented use in polymer production. The final selection includes seven commonly used bisphenol analogues from Category 1 (representing known regrettable substitutes), two chemicals from Category 2 (representing potentially safer alternatives with uncertain applications), and two biomass-based chemicals from Category 3 (representing emerging sustainable alternatives). This distribution across categories ensures our assessment covers the spectrum of current and potential future alternatives while maintaining practical constraints of data availability for exposure assessment.

# A2. Rationale for selecting the toy archetypes

The toy archetypes were selected based on four major criteria:

1. The function or category of the toy, which represents the various play behaviors in which children of specific ages engage and how they use products during play.
2. The age groups of children using toys. The selected toy archetypes are expected to cover different age groups of the entire early childhood, i.e., from 6 months to 11 years.
3. Availability of concentration data for BPA and its alternatives in the selected toy archetypes.
4. Availability of data for the toy characteristics (dimensions, material, density, weight, etc.) and toy usage (use duration/frequency, hand contact frequency, mouthing frequency, etc.)

According to these four major criteria, the toy archetypes were selected following a stepwise process, as described below.

## Step 1. BPA exposure scenarios

To develop BPA exposure scenarios, we first considered the function of the toy, which is reflected by the main toy categories defined by the CPSC [5], including 1) exploratory and practice play, 2) building play, 3) pretend and role play, 4) game and activity play, 5) sports and outdoor play, 6) educational play, and 7) media play. Second, we identified specific toys in each main toy category for 10 different age groups from birth to 12 years. Third, we searched the IC2 database and selected toys that were reported by the manufacturers to contain BPA. Combining these three types of information, we developed a total of 525 BPA exposure scenarios, provided in Appendix B, sheet “BPA_ExpScenarios”.

We then analyzed these 525 BPA exposure scenarios through the following steps:

1. Filtering exposure scenarios with a “Yes” for “BPA Determination”;
2. Counting the number of exposure scenarios for each unique combination of age group – toy category/function – toy name;
3. Filtering the unique age group – toy category/function – toy name combinations with >= 3 counts in step 2;
4. Extracting the unique toy names from the results of step 3.

This analysis resulted in 10 unique toy names as presented in Table A2.1.

Table A2.1 Unique toy names identified in Step 1

| **Toy Category/Function** | **Toy Name** | **Range of age groups covered** |
| --- | --- | --- |
| Pretend and role play | Dolls/Soft Toys (Non Powered) [10005142] | Birth to < 11 years |
| Pretend and role play | Action Figures (Powered) [10006396] | 3 to < 16 years |
| Pretend and role play | Fancy Dress Costumes/Accessories Other [10005173] | 1 to < 11 years |
| Pretend and role play | Dolls/Soft Toys (Powered) [10005143] | 2 to < 11 years |
| Building play | Toy Building Blocks (Non Powered) [10005166] | 2 to < 16 years |
| Media play | Pens [10001235] | 4 to < 16 years |
| Outdoor play | Toys – Ride-on (Powered) [10005188] | 1 to < 16 years |
| Pretend and role play | Action Figures (Non Powered) [10006395] | 3 to < 16 years |
| Pretend and role play | Necklaces/Necklets [10001090] | 3 to < 16 years |
| Pretend and role play | Practical Jokes [10005443] | 3 to < 16 years |

7 out of these 10 unique toy names belong to the toy category/function “pretend and role play”. However, for toy archetypes, we aim to select only 1-2 toys per toy category/function which cover the widest range of age groups, so we need to reduce the number of toys for “pretend and role play”. By examining these 7 toy names, we decided to keep “Dolls/Soft Toys (non-powered)” but exclude “Action Figures (powered and non-powered)” and “Dolls/Soft Toys (powered)”, because these toys are similar to each other while dolls/soft toys (non-powered) cover all age groups from birth to 11 years. Similarly, we kept “Fancy Dress Costumes” and excluded “Necklaces/Necklets” because they are similar toys while “Fancy Dress Costumes” covers most of our targeted age group (i.e., from 6 months to 11 years). “Practical Jokes” is also excluded because it is unclear what toys it refers to. Finally, we obtained the list of 5 toys as listed in Table A2.2 which covers 4 of the 7 main toy categories defined by the CPSC. The 3 toy categories that are not covered include: “Exploratory and practice play”, “Game and activity play” and “Educational play”.

Table A2.2 Toy names selected in Step 1

| **Toy Category/Function** | **Toy Name** | **Range of age groups covered** |
| --- | --- | --- |
| Pretend and role play | Dolls/Soft Toys (non-powered) | Birth to < 11 years |
| Pretend and role play | Fancy Dress Costumes | 1 to < 11 years |
| Building play | Toy Building Blocks | 2 to < 16 years |
| Media play | Pens | 4 to < 16 years |
| Outdoor play | Toys – Ride-on (Powered) | 1 to < 16 years |

## Step 2. Additional toy categories

We researched IC2 High Priority Chemicals Data System (HPCDS) and found 4 toys with components that may contain BPA alternatives: teething rings, foam building blocks, foam bath letters, and animal bath toys. Since building blocks are already included in Table A2.2, they were excluded. Among the two bath toys, foam bath letters were selected as they are more commonly found in children’s homes. Teething rings were also retained to assess mouthing exposure. Thus, this list of 4 toys was reduced to 2 toys: teething rings and foam bath letters. Adding them to the list in Table A2.2 results in 7 toys in Table A2.3. This list covers 5 of the 7 main toy categories defined by the CPSC, and the two newly added toys mainly cover the younger age groups from 3 months to 3 years.

Table A2.3 Toy names selected in Step 2

| **Toy Category/Function** | **Toy Name** | **Range of age groups covered** |
| --- | --- | --- |
| Pretend and role play | Dolls/Soft Toys (non-powered) | Birth to < 11 years |
| Pretend and role play | Fancy Dress Costumes | 1 to < 11 years |
| Building play | Toy Building Blocks | 2 to < 16 years |
| Media play | Pens | 4 to < 16 years |
| Outdoor play | Toys – Ride-on (Powered) | 1 to < 16 years |
| Exploratory and practice play | Teething rings | 3 months to < 2 years |
| Bath toy | Foam bath letters | 8 months to < 3 years |

## Step 3. Toys with available concentration data for BPA and its alternatives

We searched the literature and summarized the measured concentrations of BPA and its alternatives in various children’s products (Appendix B, sheet “BPAcontent_Literature”). We curated these data by:

1. Filtering the column “Product category” as equal to “Toy”;
2. Filtering the column “Concentration” as numbers, i.e., exclude the values such as “ND”, “<LOQ”, etc.

This curation resulted in 42 toy products. Comparing these 42 toy products to those listed in Table A2.3, we removed the toy products that are the same or very similar to the 7 toys in Table A1.3, the unique toys left include “ball”, “banana”, “apple”, “grape”, “horse”, “giraffe”, “pony”, “toy spoon” and “tiger toy”. The most common toy among these toys would be the “ball”, so we added “ball” to the list in Table A2.3.

We also searched the Interstate Chemicals Clearinghouse (IC2) database (<https://www.theic2.org/>) and extracted the concentration categories of BPA and its alternatives for various children’s products from the database. By examining these products and focusing on the toys, we found a unique toy product “Toy Vehicles – Non-ride” and added to the list above, resulting in 9 toys in Table A2.4.

Table A2.4 Toy names selected in Step 3

| **Toy Category/Function** | **Toy Name** | **Range of age groups covered** |
| --- | --- | --- |
| Pretend and role play | Dolls/Soft Toys (non-powered) | Birth to < 11 years |
| Pretend and role play | Fancy Dress Costumes | 1 to < 11 years |
| Building play | Toy Building Blocks | 2 to < 16 years |
| Media play | Pens | 4 to < 16 years |
| Outdoor play | Toys – Ride-on (Powered) | 1 to < 16 years |
| Exploratory and practice play | Teething rings | 3 months to < 2 years |
| Bath toy | Foam bath letters | 8 months to < 3 years |
| Sports play | Ball | 2 to < 16 years |
| Pretend and role play | Toy vehicles – non-ride | 1 to < 11 years |

## Step 4. Toys with availability data for characteristics and usage

We examined the data availability for the 9 toys listed in Table A2.4 in existing databases, including USEtox 3 product database and ConsExpo factsheets. We found data for dolls, building blocks, and bouncy ball in the USEtox 3product database. In ConsExpo Children’s Toys Factsheet [6], we found data for “Cowboy suit”, which is a type of “Fancy Dress Costumes”, so we used “Costume” as a toy archetype.

For “Teething rings”, “Pens”, “Foam bath letters” and “Toy vehicles”, no data were available in existing databases, so we took these toys from Dr. Lei Huang’s home and measured their relevant characteristics directly.

The “Toys – Ride-on (Powered)” is meant for outdoor play. Since the present modeling study focuses on indoor exposures, we excluded this toy from the final list.

Therefore, the final list of toy archetypes includes 8 toys, as presented in Table A2.5. This final list covers 5 of the 7 main toy categories defined by the CPSC, and covers age groups from birth to 16 years, which is expected to represent the various play behaviors in different age groups of children.

There are 2 out of the 7 main toy categories defined by CPSC not covered in our final list in Table A2.5, including “Game and activity play” and “Educational play”. In the 525 BPA exposure scenarios that we developed, the “Game and activity play” category only includes one type of toy: puzzles. However, BPA and alternatives have not been determined in puzzles, so we did not include it in the selected toy archetypes. Puzzles are generally made of carboard or wood, which are not expected to contain BPA. For the “Educational play” category, no toys in this category were identified in the 525 BPA exposure scenarios that we developed. According to CPSC, the “Educational play” category includes books and learning products such as flashcards, cameras, magnetics letters & numbers, science sets, microscopes & telescopes [5]. Books can be used by children from 4 months to 12 years old, while learning products can be used by children from 2 to 12 years. These products may contain plastic or epoxy materials that may contain BPA and alternatives. However, due to limited data availability on the characteristics, usage and BPA concentrations for these products, we did not include them in the toy archetypes.

Table A2.5 Toy archetypes selected in Step 4

| **Toy Category/Function** | **Toy archetype** | **Range of age groups covered** |
| --- | --- | --- |
| Exploratory and practice play | Teething ring | 3 months to < 2 years |
| Pretend and role play | Doll | Birth to < 11 years |
| Bath toy | Foam bath letters | 8 months to < 3 years |
| Pretend and role play | Costume | 1 to < 11 years |
| Building play | Building Blocks | 2 to < 16 years |
| Sports play | Ball | 2 to < 16 years |
| Media play | Washable marker pen | 4 to < 16 years |
| Pretend and role play | Toy vehicle | 1 to < 11 years |

## Step 5. Assignment of age groups

The toy archetypes listed in Table A2.5 can be used by children of different ages, especially in shared spaces with mixed-age users. However, for modeling purposes, we assigned a default age group of a child user for each toy archetype based on U.S CPSC guidelines [5], ensuring that the default age groups cover the entire age range from 6 months to 11 years. We mapped the CPSC age groups to the U.S. EPA age groups, since in USEtox we use child characteristics and contact frequency data from the U.S. EPA Exposure Factors Handbook [7]. The assigned default age groups are presented in Table A2.6.

Table A2.6 Final selected toy archetypes, default age groups and material types

| **Toy archetype** | **Default age group** | **1st material** | **2nd material** |
| --- | --- | --- | --- |
| Costume | 3 to <6 years | Polyethylene terephthalate (PET) | Polyamide (PA) |
| Teething ring | 6 to <12 months | Silicone rubber | Flexible polyvinyl chloride (PVC) |
| Ball | 3 to <6 years | Polyurethane (PU) | Flexible polyvinyl chloride (PVC) |
| Doll | 2 to <3 years | Flexible polyvinyl chloride (PVC) | Acrylonitrile butadiene styrene (ABS) |
| Foam bath letters | 2 to <3 years | Ethylene vinyl acetate (EVA) | Flexible polyvinyl chloride (PVC) |
| Building blocks | 3 to <6 years | Acrylonitrile butadiene styrene (ABS) | Polycarbonate (PC) |
| Toy vehicle | 6 to <11 years | Acrylonitrile butadiene styrene (ABS) | Polystyrene (PS) |
| Washable marker pen | 6 to <11 years | Polypropylene (PP) | Polycarbonate (PC) |

## Step 6. Assignment of material types

BPA is primarily used to manufacture polycarbonate and epoxy resins. Thus, generally only the toys containing polycarbonate plastics or epoxy resins would contain BPA. However, BPA may also be present in other types of plastics such as PVC and polyester [8, 9]. Thus, for each toy archetype, we assigned two material types for predicting D_m_, K_ma_ and K_mw_ which represent the most commonly used or most plausible materials for a specific toy archetype. The material type selection of each toy archetype is presented in Table A2.6 and is described below.

*Teething ring* – Currently, most teething rings are made of food-grade silicone, so we assumed “silicone rubber” as the 1^st^ material type. Another possible material type for teether is PVC, although most baby teethers are no longer made of PVC nowadays, so for the 2^nd^ material type we assigned “flexible PVC” as a comparison to silicone rubber. A 2016 study found BPA leached from all 59 teethers collected from the U.S. market but did not specify the material types of those teethers [10]. Thus, we assume that both silicone and PVC teethers may contain BPA.

*Doll* – Dolls consist of different body parts which can be made from different types of polymers. For example, the various parts of a Barbie doll are made of PVC, PVDC (poly vinylidene dichloride), ABS (acrylonitrile butadiene styrene), EVA (ethylene vinyl acetate), or PP (polypropylene) [11]. The soft and more flexible parts of a doll such as head, arms and legs are generally made of PVC, while the more rigid parts such as torso and battery lid are made of ABS [8, 11]. Studies have detected BPA in plastic dolls [8]. Thus, we assumed “flexible PVC” as the 1^st^ material type and “ABS” as the 2^nd^ for dolls.

*Foam bath letters* – most foam letters sold in the U.S. market are made from EVA foam, so we assigned the set 1 material type as EVA polymer. However, EVA generally does not contain BPA. A study by Kirchnawy et al. measured several bath toys which were made from PVC and detected BPA [8], which implies that PVC bath toys may contain BPA. Assuming that foam bath letters are similar as other bath toys, we assigned “Flexible PVC” as the set 2 material type for the foam bath letters.

*Costume* – Fancy dress costumes are mostly made from polyesters. Polyester fabric most commonly refers to polyethylene terephthalate (PET) [12], so we assigned the 1st material type as "PET”. A 2017 study found BPA and other bisphenols in polyester or cotton textiles and infant clothing [9], suggesting that polyester costumes may contain BPA. On the other hand, polyamide (PA) or Nylon is also a commonly used fabric material for clothing [13], so we assumed “PA” as the 2^nd^ material type for the costume.

*Building blocks* – Building blocks such as LEGO are mostly made from ABS, but other types of plastics are also used, such as polycarbonate (PC), polypropylene (PP), polyamide (PA), polyoxymethylene (POM), etc. [14]. In particular, PC has been used to make the vast majority of transparent parts in LEGO’s history until around 2019 [14]. Therefore, we assigned the 1st material type as “ABS” and the 2^nd^ as “PC”, since PC is the material mostly likely to contain BPA.

*Ball* – Polyurethane (PU) and PVC are common materials used to make soccer balls [15]. A 2024 study [16] tested 84 plastic toys from the Japanese market which included 4 soft plastic balls. Three of these balls were made of PVC and one was made of PU [16]. We also learned from private communications with the Chinese toy sector that PU was the typical material for bouncy play balls. Thus, we assigned the 1^st^ material type as “PU” and the 2^nd^ as “flexible PVC”.

*Washable marker pen* – The body of a marker pen is usually made of propylene and ethylene polymers [17]. We thus assigned the 1^st^ material type as “polypropylene (PP)”, but PP typically does not contain BPA. PC contains BPA but we are not sure if the body of a marker pen can be made from PC, but we still assumed the 2^nd^ material type as “PC” as a test case.

*Toy vehicle* – A 2024 study [16] tested 84 plastic toys from the Japanese market and found that rigid toy vehicles can be made of ABS, PP, PU, POM or PS (polystyrene), with ABS as the most common material. As a result, we assumed the 1^st^ material type as “ABS”. For the 2^nd^ material type, we selected “PS” to have more diverse material types across the different toy archetypes.

# A3. Modeling procedures

The modeling of children’s exposures to BPA and alternatives in toys followed a 4-step process, which is described below. All model runs in USEtox 3 were setup as a batch, which is provided in Appendix B, sheet “Batch run setup”.

## A3.1 Exposure to BPA and alternatives in individual toys

This is Step 1 of the modeling process, which aimed to:

1. Assess children’s exposure levels of BPA and alternatives in individual toys, using the default age group of each toy archetype.
2. Examine the effect of contact level and material type of toys on the exposure.
3. Assess the multi-pathway exposures and identify the dominant pathway(s) for each chemical.
4. Investigate the key parameters that determine the exposure levels and dominant exposure pathways.

**Modeling setup:**

For each of the 12 chemicals and 8 toy archetypes listed in Table 1 of the main text, we performed USEtox modeling using the default age group, two possible material types, and the average and high-end contact levels. Table A3.1 shows the setup of model runs. There was a total of $8\times1\times2\times2\times12=384$ model runs.

*Table A3.1 Setup of the 384 model runs described in Section A3.1*

## A3.2 BPA exposure by age group

This is Step 2 of the modeling process, which aimed to assess how the exposure to BPA in toys would vary with the age of children.

**Modeling setup:**

Since the aim of this step was exploring the age-related differences in exposure instead of comparing between alternatives, we used BPA as the representative chemical. We selected 3 toy archetypes to model for this step, including doll, building blocks and toy vehicle, because these 3 toys would be played by all age groups.

For each of the 3 selected toy archetypes, we performed USEtox modeling using five age groups from 6 months to 11 years with an average contact level. Only BPA was run for each model setup. Table A3.2 shows the setup of model runs, which included a total of $3\times5\times1\times1=15$ model runs.

*Table A3.2 Setup of the 15 model runs described in Section A3.2*

## A3.3 Aggregate exposure for a certain age group

This is Step 3 of the modeling process, which aimed to:

1. Estimate the aggregate exposure to target chemicals in toys in a certain age group.
2. Compare the aggregate exposure dose to toxicity data to evaluate potential health risk.

**Modeling setup:**

For estimating aggregate exposure, we selected the age group of 3 to <6 years as the most representative group for aggregate exposure assessment, since all 8 toy archetypes except teething ring would be played by this age group, the most among all age groups. The aggregate exposures for all 12 chemicals in Table 1 were estimated.

To assess the aggregate exposure, data on all toys of a child that contain the target chemicals are needed. In a previous study, we estimated that on average there are 54 kg plastic toys per child in a household in western countries [13]. For simplicity, we assumed that all 54 kg plastic toys contain the target chemicals, and allocated this weight across the 8 toy archetypes, as presented in Table A3.3. For example, we allocated 16 kg to the toy archetype “Doll”, which would correspond to about 32 dolls, or the dolls plus dolls-related accessories such as dollhouses. As another example, 16 kg of building blocks would correspond to about 2000 pieces of blocks.

When estimating the aggregate exposure, for inhalation and dermal gaseous exposures, we calculated the chemical emission to indoor air from the total 54 kg of toys (including the teething ring) and used this emission to estimate the exposures, since this 54 kg represents the collection of all toys in a child’s home which would have background emission to air even though the child is not playing with them. In contrast, for dermal contact, dust ingestion and mouthing exposures, we calculated the exposures using contact frequencies for one single toy, since a child can only play with one toy at a certain time. To estimate the dermal contact frequency of one single toy, the following calculations were performed:

1. We obtained a dermal contact frequency of 25.2 min/hr for all toys and for both hands when awake from the EPA Exposure Factors Handbook Table 7-36 [3], assuming 3 to <6 years is the same as 6-26 months.
2. We then obtained the average duration of sleep of 11.5 hr/d for 3 to <6 years from the CDC (<https://www.cdc.gov/sleep/about_sleep/how_much_sleep.html>).
3. There are 7 toy archetypes (all 8 archetypes except teething ring) that would be played by the age group 3 to <6 years, so we assumed that the dermal contact duration was evenly distributed among these 7 toys.
4. The final dermal contact frequency was calculated as: $25.2\div2\div7\times\frac{24-11.5}{24}=0.9375 (min/hr)$

The use duration of the modeling was set as 3 years (i.e., 1,095 days) which is the duration from 3 to 6 years. Mouthing exposure was assumed to be zero for children 3 to <6 years since the average mouthing frequencies for various objects are zero in USEtox 3 for this age group, as no literature data were found on the mouthing frequency of this age group. Table A3.3 shows the setup of model runs for this step. For each chemical, there were 15 model runs, and the exposure dose estimated from these 15 model runs were summed up to obtain the aggregate exposure dose. For all target chemicals, there was a total of $15\times12=180$ model runs.

To evaluate health risk, we compared the aggregate exposure dose to toxicological reference values for both cancer and non-cancer effects for toxicity. For characterizing the non-cancer effects, the ingestion reference doses (RfD) and inhalation reference concentrations (RfC) were obtained from a published database providing peer-reviewed toxicity values reported in various regulatory sources [18, 19]. For chemicals for which RfDs or RfCs were not available, we used the probabilistic RfDs and RfCs derived by Aurisano et al. [20, 21] from experimental animal data (ToxVal database) using the WHO/IPCS (World Health Organization/International Programme on Chemical Safety) framework for dose-response assessment [22, 23]. The collected RfDs and RfCs differentiate between general non-cancer and reproductive/developmental effects to account for around a factor 20 difference in severity affecting human lifetime loss [24, 25]. For dermal cancer slope factors (CSFs) and RfDs we applied route-to-route 1:1 extrapolation from ingestion exposure. Among the 12 target chemicals, only four has non-cancer toxicity data available, including BPA, BPS, BPAF and BADGE. No cancer toxicity data were available for any of the 12 target chemicals.

*Table A3.3 Setup of the model runs described in Section A3.3. Models are run for children 3 to <6 years with an average contact level.*

## A3.4 Aggregate exposure for a child during early childhood

**This is Step 4 of the modeling process, which aimed to:**

1. Estimate the aggregate exposure mass of BPA in toys for a child throughout early childhood, which is from 6 months to age 11.
2. Identify the time period that contributes most to the aggregate exposure and the dominate exposure pathway(s).

**Modeling setup:**

Since the nature of this step is exploratory instead of comparative, we used BPA as the representative chemical.

To assess the aggregate exposure to BPA in toys from age 0 to age 11, we modeled the sequential use of toys for one child. Since a child is not expected to actively play with toys before he/she can sit independently, we constructed the sequential use from 6 months to 11 years of age. As described in Section 2.6.3, for inhalation and dermal gaseous exposures, we estimated the exposures based on the BPA emission from the entire 54 kg of toys with a use duration of 3,830 days which is the duration from 6 months to 11 years. In contrast, for dermal contact, dust ingestion and mouthing exposures, we modeled a single toy, and the toys modeled differed by age group, as presented in Table A3.4. For the toy archetype that labeled with a multiplier in Table A3.4, e.g., “Marker pen * 12” for 3 to <6 years, we modeled the exposure for 1 marker pen over 90 days and then multiplied it by 12, because a marker pen was assumed to last for 3 months so there would be 12 marker pens used in a duration of 3 years from age 3 to age 6.

In Table A3.4, the dermal contact frequency and mouthing frequency of a single toy for each age group were estimated using the methods described in Section A3.3. The duration of sleep for each age group is presented in Table A3.5. The exposure dose estimated in each model run of Table A3.4 was multiplied by the body weight and use duration of the corresponding age group, and then summed up to obtain the aggregate exposure mass of a child during early childhood.

*Table A3.4 Setup of the 27 model runs described in Section A3.4. Models are run for BPA with an average contact level.*

*Table A3.5. Duration of sleep for each age group. Source:* [*https://www.cdc.gov/sleep/about_sleep/how_much_sleep.html*](https://www.cdc.gov/sleep/about_sleep/how_much_sleep.html)

# A4. Variation in BPA exposure by age


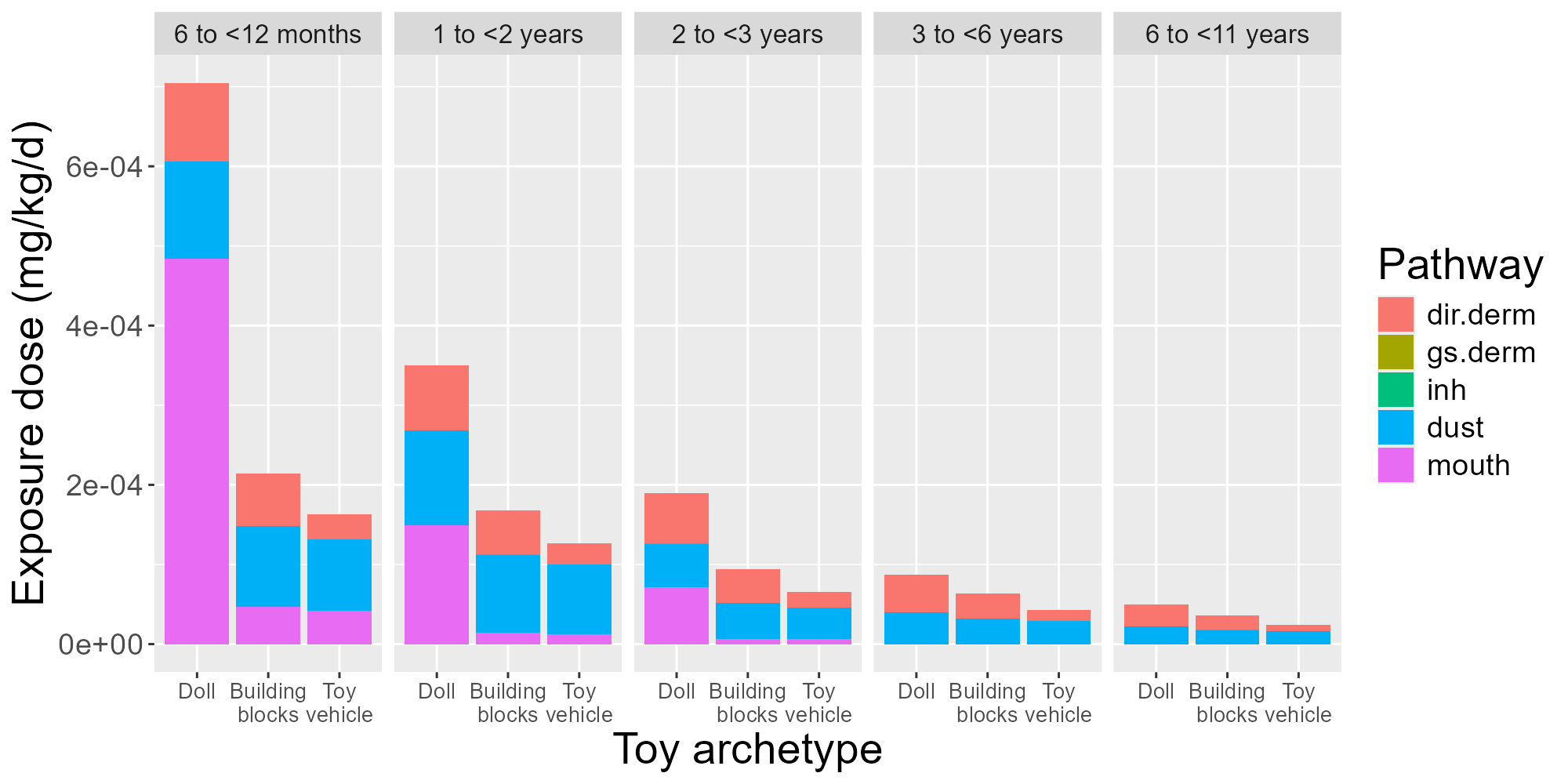


*Figure A1. Exposure dose of BPA for three toy archetypes (doll, building blocks, toy vehicles) by exposure pathway for different age groups of children.*

# A5. Chemical mass fractions and sensitivity analysis

## A5.1 Mass fraction of BPA in toys

**Literature**

In the literature, several studies have measured the migration rate of BPA from several toys to water or artificial saliva [8, 26–30], however these do not provide the mass fraction of BPA in those toys. Souza et al. reported BPA levels from 0.035 to 3.46 ppm (median: 0.43 ppm) in toys from Brazilian market [31], but these trace levels likely indicate bisphenol contamination rather than intentional use of the chemical in the toy samples. Martinez-Guijarro et al. reported BPA levels from 0.011 to 201 ppm (median: 0.32 ppm, mean: 4.89 ppm) in toys in the Middle East [32]. While most of the toys had very low BPA mass fractions indicating contamination during the manufacturing process, the toy with the maximum BPA mass fraction (201 ppm) certainly indicated intentional use. Jurikova et al. reported BPA levels from <0.05 to 625 parts per billion (ppb) in conventional and recycled textiles for adults (t-shirt and socks) [33], but these low levels likely indicated bisphenol contamination and were only slightly relevant to one toy archetype (costume).

**IC2 database**

The Interstate Chemicals Clearinghouse (IC2) database reports data on BPA mass fraction in certain toys. The IC2 database reported 36 records of BPA in toys (Appendix B, sheet “IC2 Download”). We excluded 6 records with the Concentration Category “PQL (practical quantitation limit) to less than 100 ppm” since we had no data on the PQL. We then excluded 4 records with the Concentration Category “Equal to or greater than 500 but less than 1,000 ppm” because those toy products did not fit into our 8 toy archetypes. This left us 26 records, 25 with the Concentration Category “Equal to or greater than 100 but less than 500 ppm” and 1 with the Concentration Category “Equal to or greater than 1,000 but less than 5,000 ppm”. These 26 records covered 5 out of the 8 toy archetypes, including costume, doll, building blocks, toy vehicle and marker pen. There was no specific data for the remaining 3 toy archetypes (teething ring, foam bath letters, ball).

**Summary**

Summarizing the literature data and IC2 data, we selected 300 ppm as the point estimate for BPA across 8 toy archetypes. This point estimate aimed for conservative yet central (instead of worst-case) estimates, because the majority of IC2 records indicated “100 – 500 ppm”, and 300 ppm was the average of 100 and 500 ppm. For the BPA mass fraction range used for sensitivity analysis, we assumed a uniform distribution between 50 ppm and 5,000 ppm. 50 ppm was the average between PQL and 100 ppm assuming PQL equals zero, while 5,000 ppm was the maximum mass fraction reported.

## A5.2 Mass fraction of BPA alternatives in toys

**Literature**

For BPS, Souza et al. reported levels from 0.035 to 0.26 ppm (median: 0.04 ppm) in toys from Brazilian market [31]. Martinez-Guijarro et al. only detected BPS in two toy samples from the Middle East, one at 90 ppb and the other at 25 ppb [32]. Jurikova et al. reported BPS levels from 0.277 to 2,474 ppb in conventional and recycled textiles for adults (t-shirt and socks) [33]. The BPS levels reported in these studies were all very low and likely reflect contamination instead of intentional use.

For BPF, it was not detected in textiles and toys from the Middle East [32, 33]. Souza et al. reported levels from 0.035 to 2.41 ppm (median: 0.04 ppm) in toys from Brazilian markets [31].

For other alternatives, Souza et al. reported BPZ levels from 0.035 to 0.16 ppm (median: 0.04 ppm) in toys from Brazilian markets, but did not detect any BPAF or BPAP [31]. No studies were located on the mass fraction of other alternatives in toys.

**IC2 database**

For BPA alternatives considered in this study, the IC2 database reported 3 records of BPS and 4 records of BPF in toys (Appendix B, sheet “IC2 Download”). Additionally, IC2 reported 11 records of TBBPA in toys, but this alternative is not considered in the present study. Two BPS records reported “Equal to or greater than 100 but less than 500 ppm” while one record reported “PQL to less than 100 ppm”. All 4 BPF records reported “Equal to or greater than 100 but less than 500 ppm”.

**Summary**

We assumed a point estimate of 300 ppm for BPS and BPF across all toys, since the majority of IC2 records indicated “100 – 500 ppm”, and 300 ppm was the average of 100 and 500 ppm. Since there is no available data in the literature or IC2 for the other chemical alternatives, the mass fraction of the other 9 BPA alternatives was also assumed to be 300 ppm across all toys, which aligns with regulatory "drop-in" assumptions used in analogous exposure scenarios.

For the alternatives mass fraction range used for a sensitivity analysis, we assumed a uniform distribution between 50 ppm and 500 ppm. 50 ppm was the average between PQL and 100 ppm assuming PQL equals zero, while 500 ppm was the maximum mass fraction reported.

## A5.3 Aggregate exposure of BPA and alternatives using point estimates


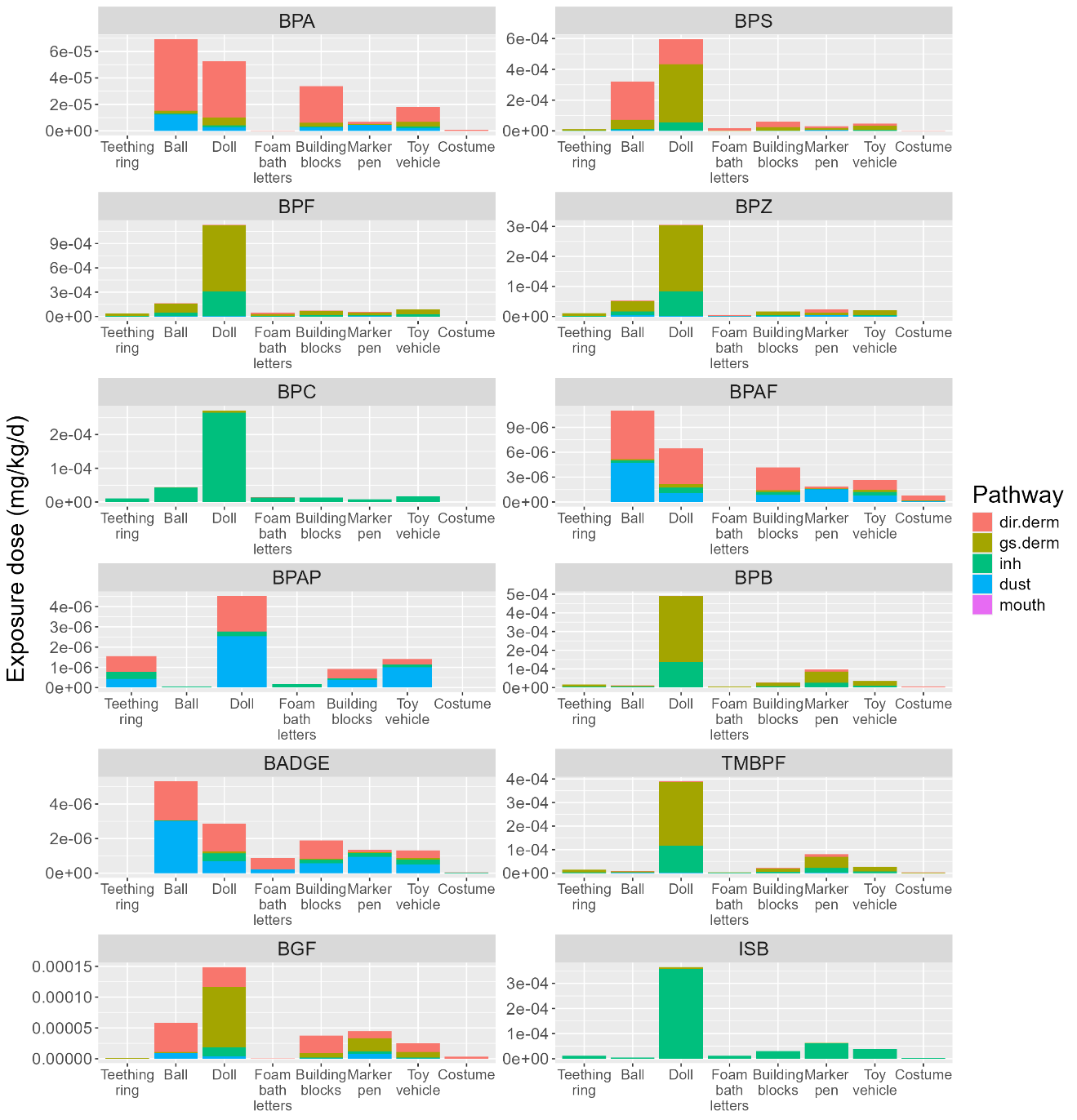


*Figure A2. Aggregate exposure to BPA and the 11 alternatives for children 3 to <6 years, showing the contribution of each toy archetype by exposure pathway.*


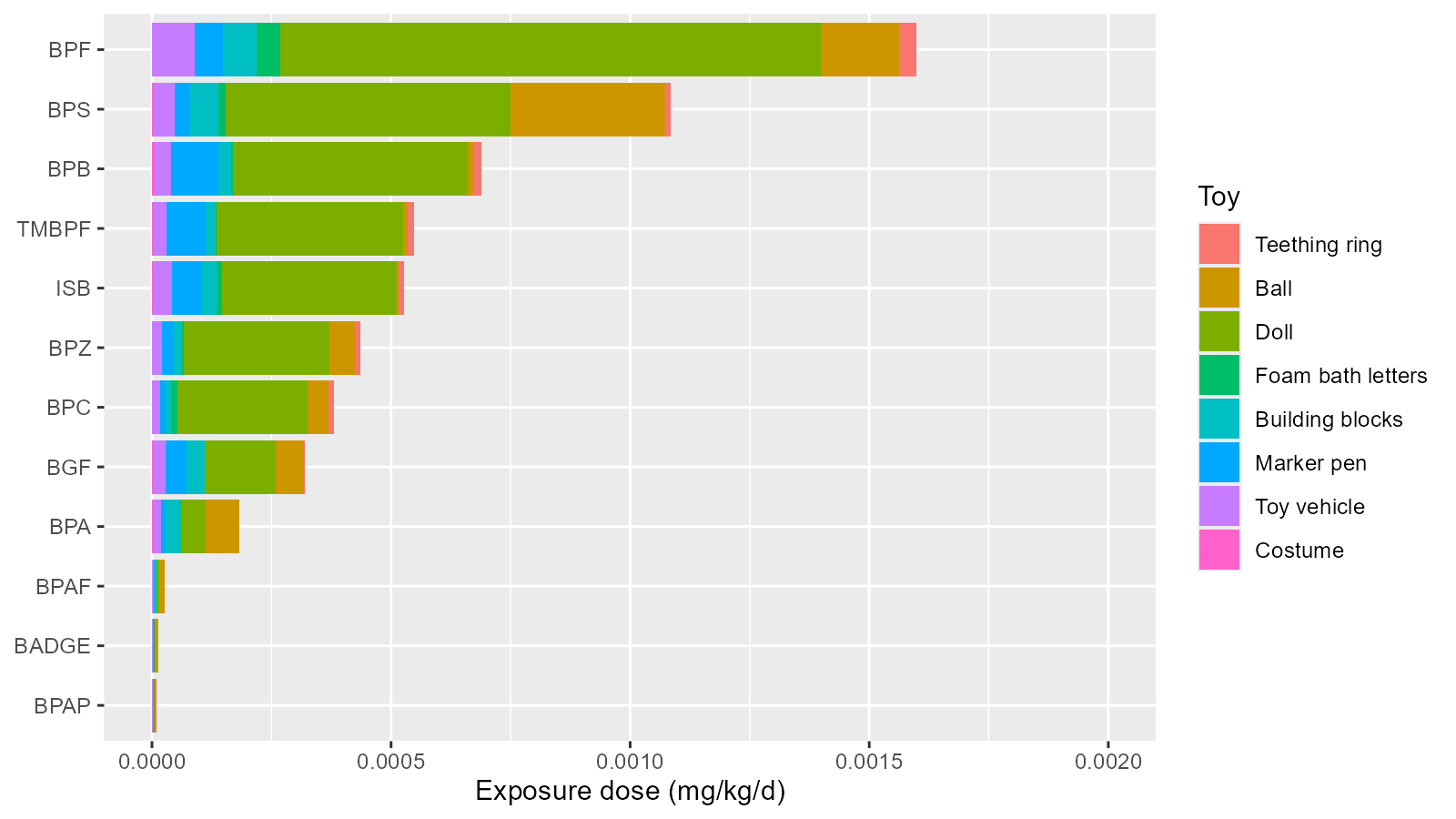


*Figure A3. Aggregate exposure to BPA and the 11 alternatives for children 3 to <6 years from the entire 54 kg toys that can be found in the home, by toy archetype.*

## A5.4 Sensitivity analysis on aggregate exposure

We conducted a sensitivity analysis to examine the effect of chemical mass fraction on the exposure estimates. As described in Sections A5.1 and A5.2, a uniform distribution between 50 and 5,000 ppm was assumed for BPA across all toys, while a uniform distribution between 50 and 500 ppm was assumed for all alternatives. The exposure estimates change linearly with the chemical mass fraction in the toy archetypes, since the exposure is calculated by multiplying the product intake fractions estimated in USEtox with the chemical’s mass fraction in the toy. Thus, the relative contribution of various exposure pathways and the variation with age are not expected to change when varying the mass fraction. However, the variation in mass fraction would certainly affect the comparison between the estimated exposures and toxicity benchmarks. As a result, we conducted the sensitivity analysis on the aggregate exposure of children 3 to <6 years to the four chemicals with available RfD data. The results are presented in Figure A4.

As shown in Figure A4, the estimated aggregate exposures of BPA via the dermal route exceeds the rep/dev RfD if its mass fraction is above 1,400 ppm, so a BPA mass fraction of 1,000 to 5,000 ppm in toys would be highly concerning. On the other hand, considering the mass fraction ranges, the estimated aggregate exposures to BPS, BPAF and BADGE were multiplied by a factor of 0.167 to 1.67, which does not exceed any RfDs even with the highest mass fraction of 500 ppm.


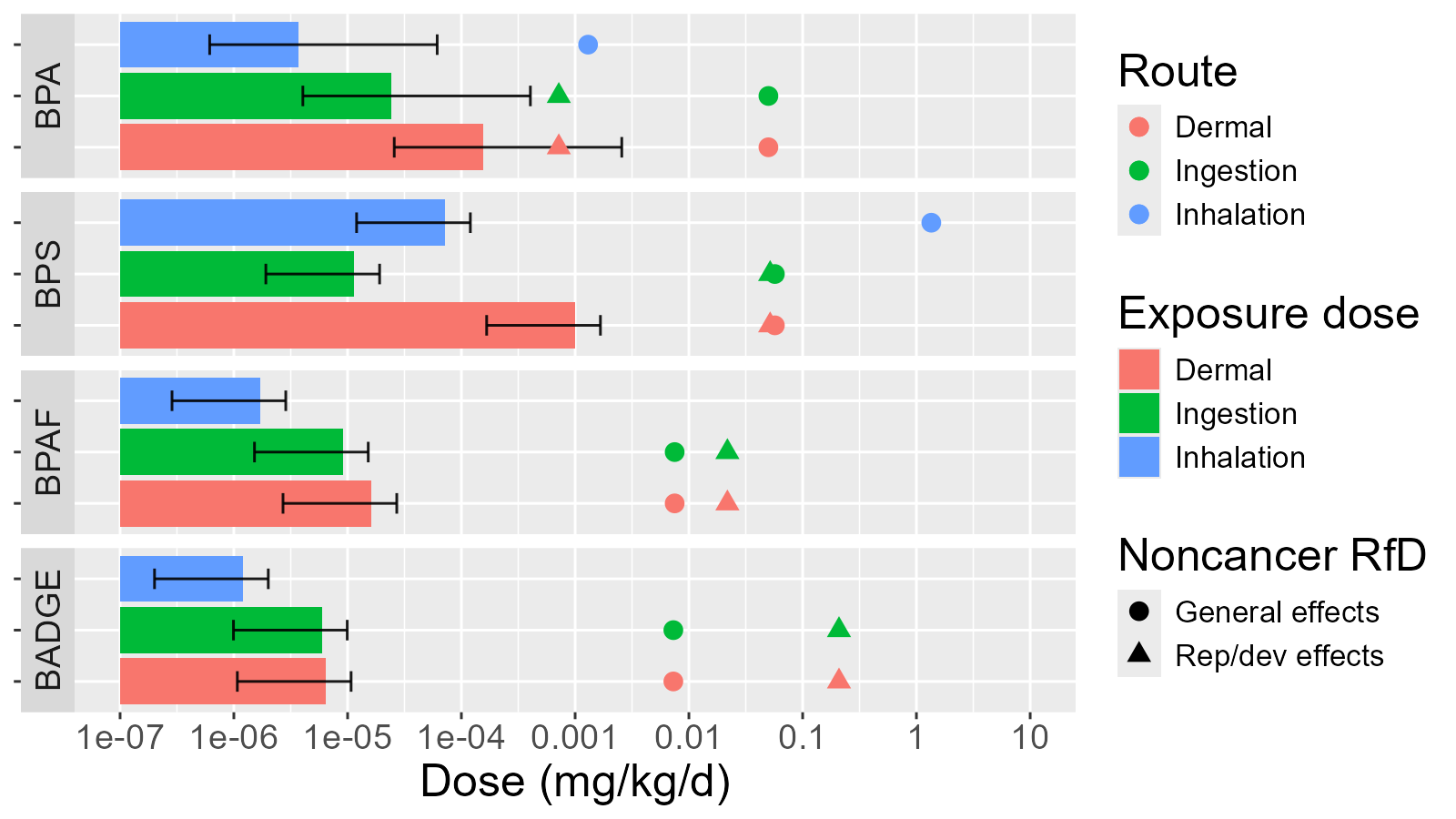


*Figure A4. Results from a sensitivity analysis showing the effect of mass fraction on the aggregate exposure of children aged 3 to <6 years to BPA, BPS, BPAF, and BADGE, with comparison to reference dose (RfD). A uniform distribution between 50 and 5,000 ppm was assumed as the mass fraction of BPA across all toy archetypes, while a uniform distribution between 50 and 500 ppm was assumed for BPS, BPAF and BADGE.*

# References

[1] ECHA. (2021). ECHA: Assessment of regulatory needs. Bisphenols. European Chemicals Agency (ECHA). Available at: https://echa.europa.eu/documents/10162/3448017/GMT_109_Bisphenols_Report_public_23502_en.pdf/1bd5525c-432c-495d-9dab-d7806bf34312?t=1647590013566. Accessed 7 Apr 2022.

[2] Stoye E. (2014). BPA substitute made from paper industry leftovers. in: Chemistry World. Available at: https://www.chemistryworld.com/news/bpa-substitute-made-from-paper-industry-leftovers/7177.article.

[3] Barrett A. (2021). Best biobased alternative for BPA. in: Bioplastics News. Available at: https://bioplasticsnews.com/2021/12/01/best-biobased-alternative-bpa/.

[4] Rooks E. (2019). Glucose derivative replaces BPA in sustainable polycarbonate plastic. in: Chemistry World. Available at: https://www.chemistryworld.com/news/glucose-derivative-replaces-bpa-in-sustainable-polycarbonate-plastic/3010964.article.

[5] CPSC. (2020). U.S. Consumer Product Safety Commission (CPSC): Age Determination Guidelines: Relating Consumer Product Characteristics to the Skills, Play Behaviors, and Interests of Children. Available at: https://www.cpsc.gov/content/2020-Age-Determination-Guidelines.

[6] Bremmer HJ and van Veen MP. (2002). RIVM: Children’s Toys Fact Sheet to assess the risks for the consumer.

[7] U.S. EPA. (2011). U.S. Environmental Protection Agency (U.S. EPA): Exposure Factors Handbook 2011 Edition (Final). Available at: https://www.epa.gov/expobox/about-exposure-factors-handbook. Accessed 19 Dec 2024.

[8] Kirchnawy C, Hager F, Osorio Piniella V et al. (2020). Potential endocrine disrupting properties of toys for babies and infants. PLOS ONE. 15(4):e0231171. doi: 10.1371/journal.pone.0231171.

[9] Xue J, Liu W and Kannan K. (2017). Bisphenols, Benzophenones, and Bisphenol A Diglycidyl Ethers in Textiles and Infant Clothing. Environmental Science & Technology. 51(9):5279–5286.

[10] Asimakopoulos AG, Elangovan M and Kannan K. (2016). Migration of parabens, bisphenols, benzophenone-type UV filters, triclosan, and triclocarban from teethers and its implications for infant exposure. Environmental Science & Technology. 50(24):13539–13547. doi: 10.1021/acs.est.6b04128.

[11] Brunning A. (2023). Life in plastic(s): The chemistry of a Barbie doll. in: Compound Interest. Available at: https://www.compoundchem.com/2023/08/18/barbie/. Accessed 19 Dec 2024.

[12] Wikipedia. (2024). Polyester. in: Wikipedia. Available at: https://en.wikipedia.org/w/index.php?title=Polyester&oldid=1258672469. Accessed 19 Dec 2024.

[13] SewingIsCool.com. (2021). Polyamide vs Polyester: 12 Differences And What Is Better. in: SewingIsCool.Com. Available at: https://sewingiscool.com/polyamide-vs-polyester-differences/. Accessed 19 Dec 2024.

[14] Schefcik D. (2022). Every Type of Plastic Used By LEGO. in: BrickNerd - All Things LEGO and the LEGO Fan Community. Available at: https://bricknerd.com/home/every-type-of-plastic-used-by-lego-5-20-22. Accessed 19 Dec 2024.

[15] Victeam Sports. (2020). What’s the Difference Between a PU, TPU and PVC Football (Soccer)? in: Victeam Sports. Available at: https://www.victeamsports.com/whats-the-difference-between-a-pu-tpu-and-pvc-football-soccer/. Accessed 19 Dec 2024.

[16] Bekki K, Eguchi A, Takaguchi K et al. (2024). Comprehensive survey on the use of plastic additives in toy products used in Japan. Environmental Health and Preventive Medicine. 29:43–43. doi: 10.1265/ehpm.24-00054.

[17] Lee L. (2015). Sharpie Life Cycle. in: Design Life-Cycle. Available at: http://www.designlife-cycle.com/sharpie. Accessed 19 Dec 2024.

[18] Wignall JA, Muratov E, Sedykh A et al. (2018). Conditional Toxicity Value (CTV) Predictor: An In Silico Approach for Generating Quantitative Risk Estimates for Chemicals. Environmental Health Perspectives. 126(5):057008. doi: 10.1289/EHP2998.

[19] Wignall JA, Shapiro AJ, Wright FA et al. (2014). Standardizing Benchmark Dose Calculations to Improve Science-Based Decisions in Human Health Assessments. Environmental Health Perspectives. doi: 10.1289/ehp.1307539.

[20] Aurisano N, Fantke P, Chiu WA et al. (2024). Probabilistic Reference and 10% Effect Concentrations for Characterizing Inhalation Non-cancer and Developmental/Reproductive Effects for 2,160 Substances. Environmental Science & Technology. 58(19):8278–8288. doi: 10.1021/acs.est.4c00207.

[21] Aurisano N, Jolliet O, Chiu WA et al. (2023). Probabilistic Points of Departure and Reference Doses for Characterizing Human Noncancer and Developmental/Reproductive Effects for 10,145 Chemicals. Environmental Health Perspectives. 131(3):037016. doi: 10.1289/EHP11524.

[22] Chiu WA and Slob W. (2015). A Unified Probabilistic Framework for Dose–Response Assessment of Human Health Effects. Environmental Health Perspectives. 123(12):1241–1254. doi: 10.1289/ehp.1409385.

[23] Chiu WA, Axelrad DA, Dalaijamts C et al. (2018). Beyond the RfD: Broad Application of a Probabilistic Approach to Improve Chemical Dose–Response Assessments for Noncancer Effects. Environmental Health Perspectives. doi: 10.1289/EHP3368.

[24] Fantke P, Chiu WA, Aylward L et al. (2021). Exposure and toxicity characterization of chemical emissions and chemicals in products: global recommendations and implementation in USEtox. The International Journal of Life Cycle Assessment. 26(5):899–915. doi: 10.1007/s11367-021-01889-y.

[25] Huijbregts MAJ, Rombouts LJA, Ragas AMJ et al. (2005). Human‐toxicological effect and damage factors of carcinogenic and noncarcinogenic chemicals for life cycle impact assessment. Integrated Environmental Assessment and Management. 1(3):181–244. doi: https://doi.org/10.1897/2004-007R.1.

[26] Negev M, Berman T, Reicher S et al. (2018). Concentrations of trace metals, phthalates, bisphenol A and flame-retardants in toys and other children’s products in Israel. Chemosphere. 192:217–224.

[27] Altannak NF and Alsaleh A. (2015). A validated uplc-uv method for bisphenola (BP-A) levels detection in importedplastic toys and drinking bottled water in Kuwait. Int J Pharm Pharm Sci. 7:351–354.

[28] Andaluri G, Manickavachagam M and Suri R. (2018). Plastic toys as a source of exposure to bisphenol-A and phthalates at childcare facilities. Environmental Monitoring and Assessment. 190(2):65. doi: 10.1007/s10661-017-6438-9.

[29] Brandsma SH, Leonards PE, Koekkoek JC et al. (2022). Migration of hazardous contaminants from WEEE contaminated polymeric toy material by mouthing. Chemosphere. 294:133774.

[30] Vicente-Martínez Y, Caravaca M and Soto-Meca A. (2020). Determination of Very Low Concentration of Bisphenol A in Toys and Baby Pacifiers Using Dispersive Liquid–Liquid Microextraction by In Situ Ionic Liquid Formation and High-Performance Liquid Chromatography. Pharmaceuticals. 13(10):301.

[31] Souza JMO, Souza MCO, Rocha BA et al. (2022). Levels of phthalates and bisphenol in toys from Brazilian markets: Migration rate into children’s saliva and daily exposure. Science of The Total Environment. 828:154486.

[32] Martínez-Guijarro K, Gevao B, Porcelli M et al. (2024). Assessment of bisphenols in children’s toy and baby products in the Middle East. Emerging Contaminants. 10(3):100374. doi: 10.1016/j.emcon.2024.100374.

[33] Jurikova M, Dvorakova D, Bechynska K et al. (2024). Bisphenols in daily clothes from conventional and recycled material: evaluation of dermal exposure to potentially toxic substances. Environmental Science and Pollution Research. 31(43):55663–55675. doi: 10.1007/s11356-024-34904-4.
